# Promoting Physical Activity in Low-Active Adolescents via Facebook: A Pilot Randomized Controlled Trial to Test Feasibility

**DOI:** 10.2196/resprot.3013

**Published:** 2014-10-30

**Authors:** Thomas R Wójcicki, Diana Grigsby-Toussaint, Charles H Hillman, Marian Huhman, Edward McAuley

**Affiliations:** ^1^Bellarmine UniversityExercise Science DepartmentBellarmine UniversityLouisville, KYUnited States; ^2^University of IllinoisDepartment of Kinesiology and Community HealthUniversity of IllinoisUrbana, ILUnited States; ^3^University of IllinoisDepartment of CommunicationUniversity of IllinoisUrbana, ILUnited States

**Keywords:** social media, physical activity, adolescents, behavior change, randomized controlled trial, feasibility

## Abstract

**Background:**

The World Wide Web is an effective method for delivering health behavior programs, yet major limitations remain (eg, cost of development, time and resource requirements, limited interactivity). Social media, however, has the potential to deliver highly customizable and socially interactive behavioral interventions with fewer constraints. Thus, the evaluation of social media as a means to influence health behaviors is warranted.

**Objective:**

The objective of this trial was to examine and demonstrate the feasibility of using an established social networking platform (ie, Facebook) to deliver an 8 week physical activity intervention to a sample of low-active adolescents (N=21; estimated marginal mean age 13.48 years).

**Methods:**

Participants were randomized to either an experimental (ie, Behavioral) or attentional control (ie, Informational) condition. Both conditions received access to a restricted-access, study-specific Facebook group where the group’s administrator made two daily wall posts containing youth-based physical activity information and resources. Primary outcomes included physical activity as assessed by accelerometry and self-report. Interactions and main effects were examined, as well as mean differences in effect sizes.

**Results:**

Analyses revealed significant improvements over time on subjectively reported weekly leisure-time physical activity (*F*_1,18_=8.426, *P*=.009, η2 = .319). However, there was no interaction between time and condition (*F*_1,18_=0.002, *P*=.968, η2 = .000). There were no significant time or interaction effects among the objectively measured physical activity variables. Examination of effect sizes revealed moderate-to-large changes in physical activity outcomes.

**Conclusions:**

Results provide initial support for the feasibility of delivery of a physical activity intervention to low-active adolescents via social media. Whether by employing behavioral interventions via social media can result in statistically meaningful changes in health-related behaviors and outcomes remains to be determined.

**Trial Registration:**

ClinicalTrials.gov NCT01870323; http://clinicaltrials.gov/show/NCT01870323 (Archived by WebCite at http://www.webcitation.org/6SUTmSeZZ).

## Introduction

### Physical Activity and Adolescents

Physical activity levels among adolescents are at all time lows [1-3], with less than one in five American adolescents meeting the recommended guidelines of accumulating at least 60 minutes of moderate-to-vigorous physical activity on a daily basis [4,5]. It is well established that increased involvement in various forms of physical activity (eg, exercise, sport, play, leisure, transportation) is associated with an array of health-related benefits including, but not limited to, physical health (eg, fat loss and musculoskeletal health), mental health (eg, reduced anxiety and improved self-esteem), cognitive health (eg, increased academic achievement), and behavioral health (eg, favorable adolescent risk profiles) [6-13].

Traditionally, physical activity interventions for youth have often used in-person, center-based modes of delivery [14,15]. Although these face-to-face interventions have been identified as the “gold standard” of behavioral therapy, they are limited in terms of reach and accessibility. Fortunately, the relatively recent introduction of the Internet into clinical practice has created several opportunities for innovative behavioral interventions [16,17]. Researchers have become increasingly more interested in using the Internet as a mode of delivery for physical activity programs [15,18], and a number of reviews attest to the feasibility and efficacy of Internet-delivered interventions for changing health behavior outcomes [19-21].

More recently, advances in Web-based programing have led to interactive communication technologies, commonly referred to as *social media*. These sites provide users with the ability to make virtual connections and interact with one another via user-generated content [22-24]. Due to the ease of use, accessibility, minimal cost, limited maintenance (on the user’s end), and various interactive communication features, these relatively new technologies have quickly gained universal acceptance [25]. As a result, researchers have begun to examine the potential of social media as a means for increasing education and social support for individual behavior change. Emerging evidence suggests that physical activity interventions using social media can be effective at influencing health and promoting behavior change [21,25-29]. Social media sites provide users with the ability for continuous self-monitoring, real-time feedback, and information exchange, all of which are conducive to behavior change [17]. Thus, further evaluation of social media as a means to influence health behaviors is warranted [30-33].

### Social Networking Sites and Adolescents

Social networking sites are one of the most popular forms of social media [27,34], especially among adolescents [32,35,36], and are becoming an alternative to email as a means for instant communication between friends [37]. Facebook, in particular, is the most widely used social networking site by adolescents or any other demographic, reaching roughly two thirds of the Internet population [38]. Adolescents, 13-17 years old, make up nearly 21% of the Facebook population [35]. Sites such as Facebook have become a prominent source of information and guidance during adolescence. For example, 57% of adolescents look to their social networking sites for advice, making them 63% more likely to do so than the typical social networker [34]. These vital communication hubs have the information and tools necessary for developing and managing healthy lifestyles, with roughly one third of online adolescents using the Internet for health-, diet-, or fitness-related information [36]. Thus, social networking sites may hold the potential to aid in the promotion of health and encourage changes in behavior [21,31-33].

### The Present Study

The *Social Media and Activity Research in Teens* (*SMART*) Trial is a social media-based intervention specifically designed to influence the physical activity behaviors of adolescents. The purpose of this innovative 8 week program was to test the feasibility of delivering a physical activity intervention to low-active adolescents over an established social media platform (ie, Facebook). Furthermore, we were interested in examining the differences in behavioral outcomes between two social media-based conditions (ie, an experimental group which received behavioral training modules vs an attentional control group). It was hypothesized that exposure to and participation in this particular intervention would provide initial support for the potential of using social media to promote positive changes in physical activity behaviors among a sample of low-active adolescents. Additionally, exposure to video-based behavioral training modules should lead to greater improvements in physical activity participation than simple exposure to physical activity-related information and resources.

## Methods

### Study Design and Participants

An 8 week randomized controlled pilot trial was designed to increase lifestyle physical activity in adolescents (NCT01870323). Participants were recruited from Champaign County, Illinois. Individuals who met inclusionary criteria were randomized (matched by age and sex) to either the intervention (ie, sharing physical activity-related content via Facebook along with weekly behavioral modules; *Behavioral Condition*) or attentional control group (ie, sharing physical activity-related content via Facebook alone; *Informational Condition*).

### Recruitment

The Internet (ie, laboratory website recruitment page, emails of former parental research participants, campus-wide listservs aimed at faculty and staff, and Facebook pages of local organizations) and targeted mailings, via the United States Postal Service, were used for recruitment purposes. Given that this trial relied heavily on parental involvement, recruitment efforts were targeted at parents or legal guardians of children between the ages of 13 and 15 years old. Advertisements for recruitment included basic information about the study, along with contact information (ie, study-specific email and website) for interested parties. To be considered for participation, a parent or legal guardian had to accompany all eligible adolescent participants. Parental guardians were screened via telephone to determine the physical activity levels of their children. Those meeting or exceeding federal guidelines were excluded. Additionally, participants were required to be English-speaking and have access to the Internet at their place of residence via a personal or family-dedicated tablet, laptop, or desktop computer. Individuals who only had mobile access to the Internet were excluded, to ensure similar user experience (although accessing the Facebook group via a mobile application was permitted). Finally, participants also had to have an active Facebook account or be willing to create one prior to enrollment.

### SMART Facebook Group

**Figure 1:**
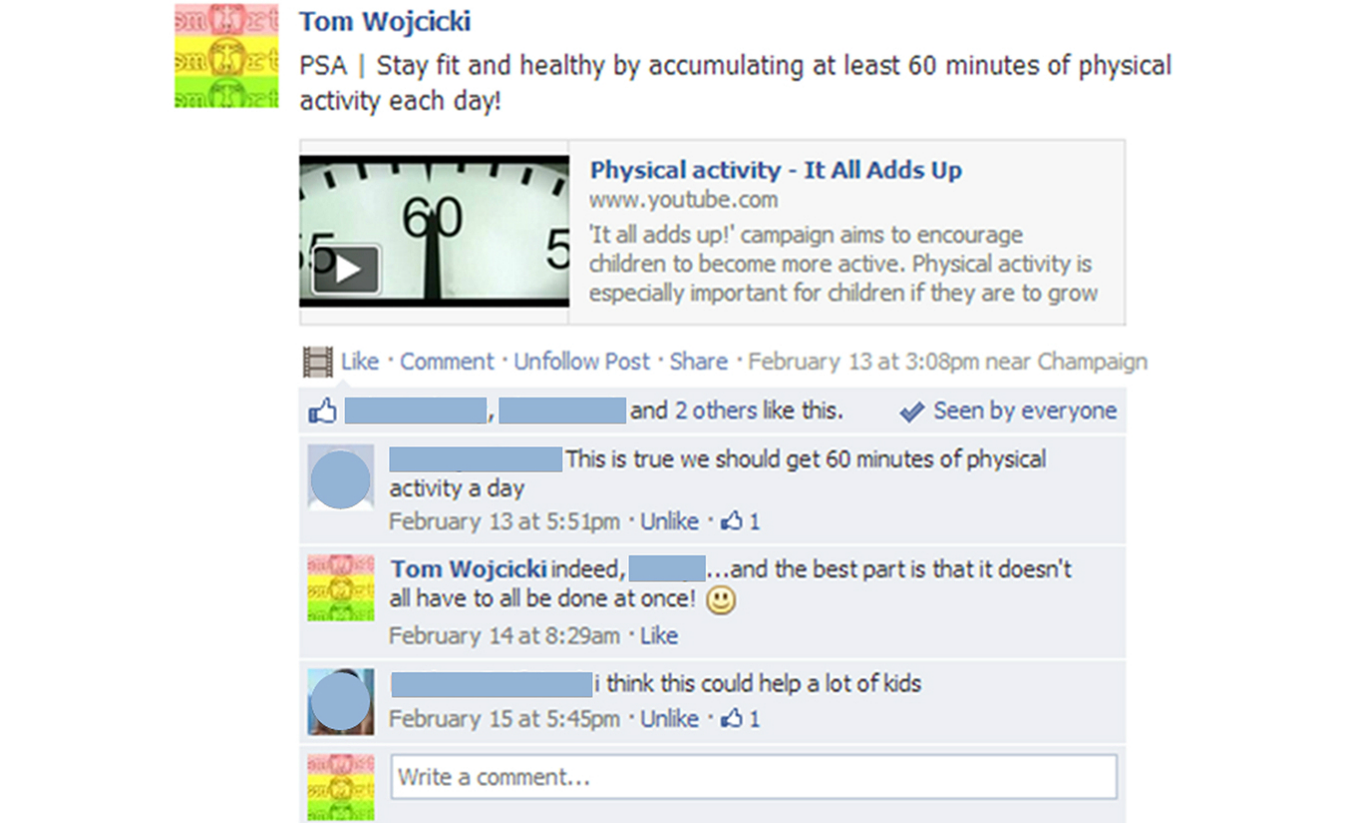
Screenshot of SMART Facebook Group post. PSA=public service announcements.

Access to a single, study-specific Facebook group (ie, the *SMART* Group) was restricted to all study participants and at least one of their legal guardians. The purpose of this group was to create a social, interactive community that revolved around the topic of physical activity for youth. Physical activity-related information and resources from around the Web were provided daily by the group’s administrator. Considering the varying characteristics and preferences of the adolescent sample, an assortment of group wall posts (ie, readily available information shared in a virtual community) were made, all of which were categorized within one of the following seven categories: (1) physical activity-related websites; (2) infographics; (3) public service announcements (PSA); (4) technology and applications; (5) local parks and facilities; (6) motivational quotations; and (7) miscellaneous topical posts. The *SMART* Group received two wall posts per day (ie, once in the morning and again in the evening), resulting in 14 total wall posts per week with equal distribution of the predefined content categories (see Table 1). In addition to these daily posts, photo albums containing physical activity campaign advertisements/posters (eg, The President’s Challenge) were uploaded and shared once per week, resulting in eight additional posts. In all, 120 posts were made over the course of the trial. Participants were encouraged to regularly view and interact with the posted content throughout the duration of the trial (Figure 1 shows posts). Recommended strategies for effective wall posts (ie, posts that encourage group-member engagement via likes and comments) were utilized throughout the 8 week program [39].

**Table 1:** Example of posted content during a typical week.

Day and time	Category	Title/“content”	Source
Monday am	Quotation	“Life is like riding a bicycle. In order to keep your balance, you must keep moving.”	Albert Einstein
Monday pm	Website	WebMD FIT Teens: Move	WebMD
Tuesday am	Infographic	The Role of Schools in Promoting Physical Activity	Active Living Research
Tuesday pm	Local	Champaign-Urbana Area Bike Routes	Champaign County Bikes
Wednesday am	Mobile app	Walking Paths App	American Heart Association
Wednesday pm	Video PSA	Sedentary-2012	American Academy of Orthopaedic Surgeons
Thursday am	Miscellaneous	We Need More Physical Education in Schools	SPARK
Thursday pm	Website	TeensHealth: Nutrition & Fitness Center	Nemours
Friday am	Local	Hiking in East-Central Illinois	Illinois Department of Natural Resources
Friday pm	Video PSA	ParticipACTION 2012: Driveway	ParticipACTION
Saturday am	Quotation	“Success isn’t how far you got, but the distance you traveled from where you started.”	Steve Prefontaine
Saturday pm	Infographic	Children and Nature: Being Active in Nature Makes Kids Healthier	National Environmental Education Foundation
Sunday am	Mobile app	Instant Heart Rate App	Azumio
Sunday pm	Miscellaneous	Piano Stairs	The Fun Theory

### Behavioral Condition

Participants in the Behavioral condition received full access to content posted on the *SMART* Group wall and were encouraged to regularly view and interact with the posts. In addition to group access, participants in the Behavioral condition regularly received study-specific behavioral modules via Facebook Messages in the form of 5-10 minute YouTube videos. These video-based modules focused on key elements of physical activity and theoretically based strategies for increased participation among youth. Over the course of the intervention, the group administrator privately delivered eight unique modules to each participant and corresponding guardian on a weekly basis (see Table 2). Along with a link to each weekly module, these messages included a personalized greeting and written information regarding the content of the module.

**Table 2:** List and location of weekly behavioral modules.

Week number	Module topic
1	Getting Started with the SMART Program
2	Physical Activity Definitions and Benefits
3	Physical Activity Guidelines
4	Goal-Setting for Physical Activity
5	Individual Expectations and Physical Activity
6	Social Support for Physical Activity
7	Overcoming Barriers to Physical Activity
8	Maintaining a Physically Active Lifestyle

### Informational Condition

Participants in the Informational condition also received full access to the *SMART* Group and were similarly encouraged to regularly view and interact with the content posted on the group wall. The group administrator via a private Facebook Message also contacted participants once a week. The frequency of these fairly generic messages (ie, weekly greetings) occurred once per week, in unison with the delivery of the behavioral modules in the experimental condition.

### Baseline Visit

During their baseline visit to the laboratory, participants, and at least one of their legal guardians, were added to the private *SMART* Group by the group’s administrator. Participants and their guardians had to first accept a “Friend Request” from the administrator in order to be electronically invited to join the *SMART* Group. To ensure confidentiality and improve experimental rigor, this group was kept private and was restricted to the randomized child-parent pairs. Once accepted to the *SMART* Group, all participants, regardless of treatment allocation, were asked (via Facebook Messages) to complete a Web-based battery of questionnaires, prior to the official start date of the trial.

The baseline laboratory visit also included assessments of participants’ height and weight. Once these data were obtained, participants were provided with an accelerometer to wear over the course of the following week. A corresponding log to validate wear time and a self-addressed, prestamped envelope to mail back the device were also provided during this visit. Finally, prior to leaving the baseline appointment, all participants, regardless of group allocation, were given pedometers to use over the course of the trial to aid in self-monitoring and behavioral regulation. Following the completion of all baseline data, participants were matched by age and sex and randomized into either the Behavioral or Informational treatment conditions.

### Measures

Assessments were conducted at baseline and at the end of the trial (ie, Week 8). All self-report assessments were administered electronically via a Web-based survey service (ie, SurveyMonkey; Palo Alto, CA, 2012). When appropriate, participants received a Web link to a battery of questionnaires via Facebook’s direct messaging service. Submitted data were unidentifiable and stored in a secure, password protected online database.

### Demographics

Demographic information obtained for the adolescent participants included sex, age, grade level, race, ethnicity, number of siblings, annual household income, and involvement in free or reduced price lunch programs. Parental guardians provided this information during the screening process, and also reported their living situation, highest level of education, and current employment status.

### Anthropomorphic Measures

Height and weight were assessed in the laboratory and measured to the nearest 0.1 inches and 0.1 pound, respectively, by using a digital column scale with stadiometer (model 736; Seca, Hamburg, Germany). From these values, participants’ body mass index (BMI) was calculated and then interpreted using age-specific BMI percentiles to classify weight status [40].

### Objective Physical Activity

Accelerometry was used to objectively assess participants’ physical activity levels. Specifically, the rechargeable, lithium-powered Actigraph accelerometer (models GT1M and GT3X; Health One Technology, Fort Walton Beach, FL) was used for this purpose, as it is the most commonly used accelerometer in the field of physical activity-related research [41], and has been shown to provide reliable and valid estimates of energy expenditure and activity levels in youth [42-44]. Participants were instructed to wear the accelerometer on their nondominant hip during all waking hours (with the exception of water-based activities) for seven consecutive days, as recommended by Trost et al [45]. Recommended cut points for predicting physical activity in youth were utilized to properly identify the amount of engagement in sedentary, light, moderate, and vigorous activities [41].

### Subjective Physical Activity

Self-reported involvement in physical activities was collected and assessed using the Godin Leisure Time Exercise Questionnaire (GLTEQ) [46]. The GLTEQ is a well validated brief assessment of usual leisure-time exercise habits [47]. Participants were asked to report how many times they participate in strenuous (ie, heart beats rapidly), moderate (ie, not exhausting), and mild (ie, minimal effort) activities for more than 15 minutes over the course of a typical week (ie, seven day period). The reported frequencies of strenuous, moderate, and mild activities are multiplied by 9, 5, and 3 metabolic equivalents, respectively, and then summed to provide a reliable estimate of total and moderate-to-vigorous weekly leisure-time physical activity.

### Sedentary Behaviors

The Adolescent Sedentary Activity Questionnaire [48] was used to assess average weekly sedentary behaviors outside of school. Questions about activities normally done while sitting or lying down during a typical week and weekend were asked, and included activities such as “Watching TV”, “Using the computer for doing homework”, and “Sitting around (chatting with friends/on the phone/chilling)”. Participants were asked to report their average time spent engaging in each of these activities for each day of the week.

### SMART Facebook Group Usage

Creators and administrators of Facebook groups are provided with basic data regarding how many (and which) group members viewed each post. Following the completion of the trial, the frequency in which each participant viewed the posted content on the *SMART* Group wall was summed and then divided by 120 (ie, total wall posts over the 8 week program) to determine the percentage of content viewed. Additionally, the rate of engagement (ie, the frequency of likes, comments, and shares divided by 120) was immediately calculated following the 8 week program.

### Program Evaluation and Feedback

Following the completion of the trial, participants completed an evaluation form regarding the strengths and weaknesses of the *SMART* Trial. There were eight questions regarding participant experience in the *SMART* Trial that were evaluated on 5-point Likert scales. Questions included: (1) “How would you rate your overall experience with the *SMART* Program?”; (2) “How interesting was the content posted on the *SMART* Group wall?”; (3) “How useful was the content posted on the *SMART* Group wall?”; (4) “On average, how many times per week did you visit the *SMART* Group?”; (5) “On average, how often did you interact with the posted content?”; (6) “How much did you learn about physical activity from this program?”; (7) “To what degree did your participation influence your physical activity?”; and (8) “How would you rate your interactions with program staff on Facebook and in person?”. Participants were also asked to rate each category of wall posts in order starting with their most favorite (ie, 1) to their least favorite (ie, 7).

### Statistical Analysis

Data were checked for missing items, normality, outliers, and errors. A series of independent-samples *t* tests were conducted between groups to identify significant differences in demographic data and descriptive statistics of study variables at baseline. Using an intent-to-treat approach, the potential of the intervention in producing behavioral changes was examined using a 2 (condition, Behavioral vs Informational group) by 2 (time) repeated measures design from data collected at baseline and at the end of the intervention. Interactions and main effects were examined, as well as mean differences in effect sizes. Due to the pilot nature of this trial and the importance of adequately powering subsequent trials, effect sizes (ie, Cohen’s *d*) were calculated to further examine the potential value of using social media to successfully deliver a physical activity intervention.

### Ethics and Informed Consent

A university Institutional Review Board (Urbana, IL, USA; Protocol No. 13019) approved the study protocol. Upon meeting eligibility criteria, participants received a letter inviting them to participate, a detailed map and information regarding their baseline appointment, and informed consent and assent documents (along with copies of both forms for personal records). At least one parental guardian was required to provide written consent to allow their child to participate in the trial, and the adolescent participant(s) were asked to read and sign an informed assent document prior to being randomized. These documents were similar in language and content and included: (1) a concise overview of the trial and its purpose; (2) an explanation of group randomization and condition-specific expectations; (3) information regarding scheduled onsite appointments, including a description of assessments to be performed at both baseline and follow-up; (4) statements regarding the potential risks and benefits associated with participation in a physical activity trial; (5) a section highlighting participants’ rights and privacy, which ensures confidentiality and stresses the voluntary nature of study involvement; (6) confirmation that participation in the study is free; and (7) contact information for the principal investigator and study staff, as well as the university’s Institutional Review Board.

Due to the sensitive nature of conducting research on adolescents, particularly in a Web-based setting, several additional steps were taken to safeguard the rights and welfare of this group. First, all identifiable information was kept in a secure, password protected database and locked filing cabinet separate from participant data, which were coded and aggregated. Second, during the screening process, the legal guardians of all potential participants were required to complete a health history questionnaire, as well as a preparticipation health screening form to ensure safe participation of each child. These procedures allowed us to screen out individuals whose physical condition contraindicates involvement in a physical activity program (n=0). Next, once eligibility was determined and consent was provided, both the adolescent participants and their legal guardians were invited to join the restricted access, study-specific Facebook group via the group’s administrator (ie, study staff). Prior to the official start of the program, the only content that was posted on the group wall were links to Facebook’s Family Safety Center and Google’s Safety Center for Families. These sites provided parent-child dyads with the necessary information and tips needed for using both Facebook and the Internet safely and appropriately. Finally, information shared on the personal Facebook profiles of participants and guardians was not collected nor distributed by the research staff for any purpose whatsoever.

## Results

### Recruitment and Study Flow

**Figure 2:**
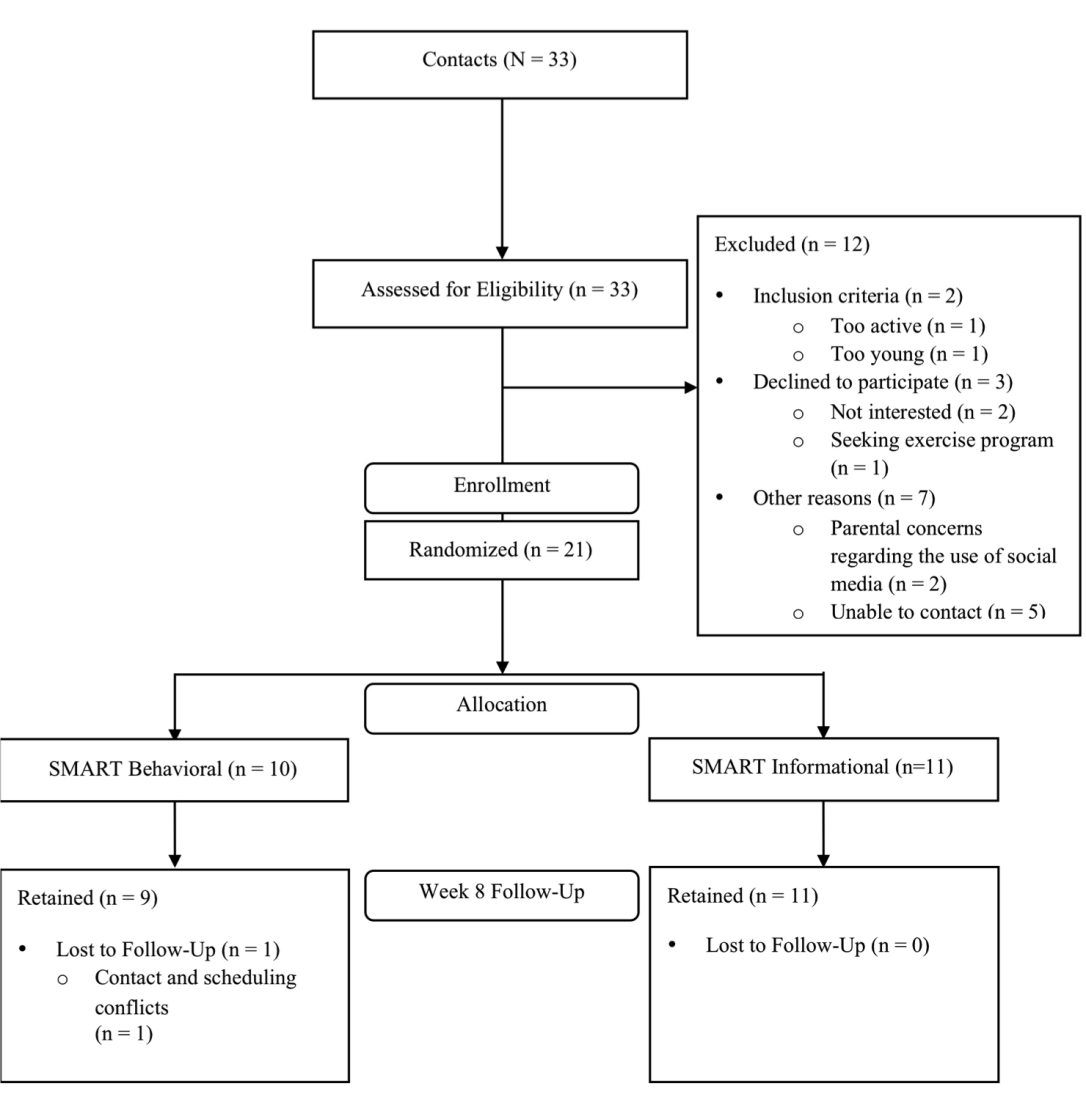
SMART Trial CONSORT diagram.

In total, 33 contacts were made and screened for eligibility, but only 21 participants met the inclusion criteria. To ensure equal allocation of subgroups to each treatment condition, eligible participants were randomized after blocking on sex and age (ie, potential confounders) via SPSS version 22 (SPSS IBM). Of the 21 randomized participants, 20 completed assessments at both baseline and follow-up, resulting in 4.8% rate of attrition (Figure 2 shows this).

### Sample Characteristics

Participant characteristics for the full study sample as well as each condition are presented in Table 3. Briefly, a majority of the sample was white (13/21, 61%), female (11/21, 52%), 13 years of age (13/21, 61%; estimated marginal mean age 13.48), in middle school (16/21, 76%), clinically overweight or obese (12/21, 57%), resided with siblings at home (20/21, 95%), and lived in a household with annual income of greater than US $100,000 (15/21, 71%). Additionally, parental characteristics revealed that (21/21) 100% of the sample resided with their mothers, a majority of whom had a bachelor’s degree or higher (17/21, 81%) and were employed full time (14/21, 66%). A series of independent-samples *t* tests revealed no significant differences in demographic variables between groups for either the participants or their legal guardians.

**Table 3:** Participant characteristics.

		Total sample	Behavioral group	Informational group
Variables		N=21, n (%)	n=10, n (%)	n=11, n (%)
**Sex**				
	Male	10 (48)	6 (60)	4 (36)
	Female	11 (52)	4 (40)	7 (64)
**Age (years)**				
	13	13 (62)	6 (60)	7 (64)
	14	6 (29)	3 (30)	3 (27)
	15	2 (9)	1 (10)	1 (9)
**Year in school (grade)**				
	6th	1 (5)	0 (0)	1 (9)
	7th	6 (29)	3 (30)	3 (27)
	8th	9 (43)	5 (50)	4 (37)
	9th	3 (14)	1 (10)	2 (18)
	10th	2 (9)	1 (10)	1 (9)
**BMI classification**				
	Underweight	1 (5)	0 (0)	1 (9)
	Healthy weight	8 (38)	5 (50)	3 (27)
	Overweight	3 (14)	1 (10)	2 (18)
	Obese	9 (43)	4 (40)	5 (46)
Siblings		20 (95)	10 (100)	10 (90)
**Race**				
	White	13 (62)	6 (60)	7 (64)
	Black	1 (5)	1 (10)	0 (0)
	Asian	2 (9)	0 (0)	2 (18)
	Biracial	5 (24)	3 (30)	2 (18)
Latino		2 (9)	1 (10)	1 (9)
**Annual household income**				
	US $10-40 K^a^	4 (19)	3 (30)	1 (9)
	US $41-70 K^a^	1 (5)	0 (0)	1 (9)
	US $71-100 K^a^	1 (5)	1 (10)	0 (0)
	US >$100 K^a^	15 (71)	6 (60)	9 (82)
Free/reduced price lunch		2 (9)	1 (10)	1 (9)

^a^ K=thousand

### Intervention Effects on Physical Activity

Independent-samples *t* tests were conducted for the descriptive variables to identify significant differences between the Behavioral and Informational conditions at baseline. At baseline, the only variable that was found to be significantly different between groups was the average amount of time spent engaging in vigorous leisure-time physical activity, where the Informational condition reported a higher rate of engagement (mean 42.55, SD 21.45) in comparison to the Behavioral condition (mean 37.60, SD 15.11); *t*_17_= -2.35, *P*=.03.

Next, a series of mixed model analysis of variance (ANOVAs) were performed using a 2 (treatment, Behavioral and Informational conditions) by 2 (time, baseline and Week 8) repeated measures design to examine the effectiveness of the intervention in producing changes in physical activity behaviors (see Table 4). Analyses revealed that there were significant improvements over time on subjectively reported weekly leisure-time physical activity, but there was no interaction between time and condition. Changes in subjectively reported moderate-to-vigorous physical activity approached significance over time, but, again, significant interaction effects were not found. Furthermore, there were no significant time effects among the objectively measured physical activity variables, including average daily minutes spent being physically active, sedentary time, moderate-to-vigorous physical activity, and total physical activity. Similarly, significant group by time effects were not present for any of the objectively assessed variables. Finally, analyses of self-reported time spent engaging in weekday and weekend sedentary behaviors did not produce significant time or interaction effects.

Given the small sample size of this trial, effect sizes (ie, Cohen’s *d*) were calculated to identify the patterns of change for the total sample and within each treatment group. Mean values for physical activity and sedentary outcomes as well as effect sizes are reported in Table 5. Changes in the behavioral outcomes were generally moderate in size and in the expected direction.

**Table 4:** Time and interaction effects for behavioral outcomes.

			Time effects				Time X group effects			
Variables			*M* ^a^	*F*_1,18_	*P*	*η* ^2^	*M* ^a^	*F*_1,18_	*P*	*η* ^2^
**Subjective physical activity**										
		MVPA^b^ leisure-time	47.250	4.186	.056	0.189	46.949	0.029	.868	0.002
		Total leisure-time	57.900	8.426	.009	0.319	57.465	0.002	.968	0.000
**Objective physical activity**										
		MVPA^b^ counts	179.539	0.649	.435	0.048	179.199	1.045	.325	0.074
		Total counts	279,923.933	1.493	.243	0.103	279,238.415	0.631	.441	0.046
**Sedentary behavior**										
		Weekday	56.464	0.445	.513	0.024	56.744	0.118	.735	0.007
		Weekend	65.838	0.068	.798	0.004	65.881	0.057	.814	0.003

^a^Estimated marginal means

^b^MVPA = moderate-to-vigorous physical activity

**Table 5:** Descriptive statistics and effect sizes of study variables.

Variables		Baseline	Follow-up	Effect size Cohen’s *d*
		mean (SD)	mean (SD)	
**MVPA** ^a^ **leisure-time**				
	Behavioral	37.60 (15.11)	50.22 (14.08)	0.86
	Informational	42.55 (21.45)	57.36 (37.79)	0.50
	Total sample	40.19 (18.42)	54.15 (29.12)	0.59
**Total leisure-time**				
	Behavioral	43.00 (17.66)	62.56 (17.21)	1.12
	Informational	52.64 (21.50)	71.00 (34.65)	0.65
	Total sample	48.05 (19.90)	67.20 (27.84)	0.80
**MVPA** ^a^ **counts**				
	Behavioral	152.55 (33.30)	189.74 (53.73)	0.85
	Informational	177.69 (83.73)	182.45 (66.51)	0.06
	Total sample	165.71 (64.57)	185.85 (58.85)	0.33
**Total counts**				
	Behavioral	235,884.95 (56,843.58)	293,563.02 (86,377.37)	0.81
	Informational	276,452.27 (119,017.51)	294,741.53 (108,012.65)	0.16
	Total sample	257,134.50 (94,697.62)	294,191.56 (95,033.31)	0.39
**Sedentary weekday**				
	Behavioral	61.18 (21.39)	57.68 (26.02)	-0.15
	Informational	54.54 (19.22)	53.34 (20.54)	-0.06
	Total sample	57.70 (20.05)	55.30 (22.63)	-0.11
**Sedentary weekend**				
	Behavioral	67.65 (38.01)	65.25 (34.85)	-0.07
	Informational	65.49 (19.68)	65.40 (22.19)	0.00
	Total sample	65.12 (29.07)	65.33 (27.76)	0.01

^a^MVPA = moderate-to-vigorous physical activity

### Program Engagement and Feedback

Following program completion, participant use and engagement were assessed. Briefly, (96/120) 80.0% of the daily posts made on the *SMART* Group wall were viewed by the total sample. This rate was notably higher in the Behavioral condition (104/120, 86.6%) than in the Informational condition (88/120, 73.3%), however this difference was not statistically significant. Group engagement (ie, likes, comments, and shares), on the other hand, was relatively low among the total sample (32/120, 26.7%), and similar between the Behavioral and Informational conditions (ie, 33/120, 27.5% and 31/120, 25.8%, respectively).

Participants were asked to anonymously complete a program evaluation and feedback form that was designed specifically for this trial. There were 20 of the 21 participants completed these forms at their follow-up appointment. Results revealed that (14/20) 70% of the study sample was “satisfied” to “very satisfied” with their overall experience with the *SMART* Trial. Additionally, (11/20) 55% of the sample found the content posted on the *SMART* Group wall to be “interesting” to “very interesting”, and (9/20) 45% found the posted content to be “useful” to “very useful”. On average, (11/20) 55% of the participants visited the *SMART* Group “1-2 times per week”, while (9/20) 45% interacted with the posted content “1-2 times per week”, as well. Furthermore, (10/20) 50% of those surveyed indicated that they learned “a good amount” to “a great deal/a lot” about physical activity from their involvement in this program, and (14/20) 70% felt that their participation in the program influenced their physical activity “some” to “a good amount”. And finally, when asked to rate their experiences with study staff (over Facebook and in person), (19/20) 95% of the participants reported having “good” to “excellent” interactions. Insight into participants’ preferred categories of wall posts was also assessed, with the Technology/App category being the most preferred type of shared content, while Infographics were rated as the least preferred category (see Table 6).

**Table 6:** Average ratings of wall post categories.

Category	Mean^a^ (SD)
Infographics	4.84 (2.39)
Local	4.26 (1.63)
Quotation	3.89 (1.82)
Technology	2.89 (1.88)
Video PSA	4.58 (2.09)
Websites	3.05 (1.78)
Miscellaneous	3.79 (1.87)

^a^ Lower means indicate more favorable ratings

## Discussion

### SMART Trial Objectives

The purpose of the *SMART* Trial was to examine the feasibility of using social media to deliver a physical activity intervention to low-active adolescents 13 to 15 years old. An additional aim of this trial was to examine the degree of changes in behavioral outcomes within the whole sample, as well as both treatment conditions.

### Primary Outcomes

According to results from the ANOVAs, participation in total leisure-time physical activity was the only behavioral outcome to positively and significantly change over time. Furthermore, significant group by time interactions were not present for any of the behavioral outcomes assessed. Despite the lack of statistically significant findings, however, involvement in this trial did result in small increases in objectively assessed moderate-to-vigorous and total physical activity, as well as time spent being physically active. Similarly, subjectively assessed physical activity resulted in improvements, but the effects were larger for moderate-to-vigorous physical activity (MVPA) and total weekly leisure-time physical activity. Although both forms of measurement are intended to capture one’s level of physical activity, it is important to note that the GLTEQ was specifically designed to assess *leisure-time* physical activity behaviors [46], whereas the accelerometer assesses *all* physical activity accumulated throughout the day, regardless of activity type. Therefore, participation in this trial appeared to be more beneficial for influencing leisure-time physical activities, such as going to the park or walking the dog, than for total daily physical activities, such as getting ready for school or doing chores.

Due to the lack of statistical power to reveal conventional differences between treatment conditions, effect sizes were calculated to further examine changes in the behavioral outcomes. Effect sizes for both the subjectively (ie, MVPA and total leisure-time physical activity) and objectively (ie, MVPA and total counts) assessed physical activity variables were, in most cases, moderate to large in size and in the hypothesized direction for the study sample. Conversely, changes in self-reported sedentary behaviors were small to nonexistent. This is not entirely surprising, however, as the purpose of this trial was not aimed at minimizing sedentary behaviors, but, rather, increasing regular participation in physical activities.

It should be noted that the effect sizes for physical activity in this particular trial were generally larger than those reported in the literature. For example, Kamath et al conducted a meta-analysis on behavioral interventions aimed at improving physical activity levels in children and adolescents, noting that youth-based physical activity interventions typically result in a significant, but small effect (*d*=0.12; range, 0.04-0.20) [49]. Moreover, Lau et al conducted a review of information and communication technology-based interventions and also found small effect sizes, ranging from 0.03 to 0.41, always favoring the intervention over the control conditions [24]. However, none of the interventions in these reviews were entirely delivered via social media. The social and interactive nature of the *SMART* Trial, in conjunction with the weekly receipt and viewing of the video-based behavioral modules, may provide some insight as to why this social media-delivered intervention resulted in larger effects than similar programs, which have preceded it.

Although these results appear to be promising, they should be interpreted with caution. The effect size results are helpful in determining the degree of influence that program involvement may have had on physical activity behaviors, but there remained an inability to detect significant differences between the treatment conditions via inferential statistics. Moreover, the limited power and lack of a true control group (ie, not receiving any form of intervention throughout the study period) limits the ability to adequately assess whether or not the addition of the behavioral modules, in particular, increased the efficacy of this social media-delivered intervention and contributed to changes in physical activity. The changes that occurred in the total study sample or by condition could have also been the result of many other factors including, but not limited to, the provision of pedometers, parental support, and allotted screen time. At the very least, results from these secondary analyses provide additional support for the need to further investigate this novel approach of delivering behavioral interventions via social media.

Finally, the design and delivery of the *SMART* Trial resulted in a relatively high rate of program engagement among study participants in both treatment conditions. This may have been the result of continually changing, yet focused content shared in a virtual community of similar others, as well as following guidelines for effective wall posts [39]. Furthermore, participants were given blatant instructions and reminders to regularly view and interact with the posted content on a daily basis. It should be noted, however, that passive engagement (ie, viewing shared content) was greater than active engagement (ie, likes, comments, and shares). This unique finding warrants further investigation, as passive engagement may simply serve as an indicator of program adherence, whereas active engagement may be indicative of participants’ interests in and preferences for select content. Whether or not these types of virtual interactions can translate to meaningful, real-world changes in health behaviors, such as physical activity, remains to be determined.

### Strengths

To our knowledge, this is the first pilot trial examining the feasibility of delivering a randomized controlled physical activity intervention entirely via social media, and specifically targeting the understudied demographic of early adolescence. The use and comparison of objective and subjective assessments of physical activity also reflect strengths of the study, as multiple forms of measurement can provide greater accuracy of and insight to physical activity behaviors among youth [50]. Finally, participants reported a high degree of satisfaction with their participation in this program, indicating that the posted content was interesting, informative, and useful. Therefore, delivering a behavioral intervention over social media appears to be a practical and well accepted approach to encouraging youth to become more physically active.

### Limitations

Several limitations with this trial should be considered when interpreting the results and building upon the findings. First, despite considerable efforts in trying to recruit participants for this trial, the study sample was relatively small. A larger sample size would improve the power of the study and potentially reduce the amount of variance among reported outcomes. Additionally, a majority of the sample came from higher socioeconomically status households. Whether or not similar results would be found in participants from lower socioeconomic households remains to be determined. A further limitation was the lack of ability to track the viewership of the weekly behavioral modules by participants (ie, the only difference between the two treatment conditions). Examination of the compliance and frequency of viewership could have provided a more concrete conclusion regarding the overall utility and worth of these modules in promoting positive changes in physical activity behaviors. Finally, issues regarding recall and reporting biases should be taken into consideration when interpreting results obtained from subjective assessments.

### Future Directions

Research utilizing and examining the effectiveness of social media in improving health behaviors and outcomes is still in its nascent stage and requires further investigation [51]. While the larger than average effect sizes found in this pilot trial are encouraging, larger studies should be conducted to better determine the effectiveness of using social media to promote physical activity behaviors in low-active adolescents. If efficacy can be established, similar trials should be conducted and evaluated with other populations of varying ages and perhaps even disease states. It is also important to examine participants’ abilities to maintain improved levels of physical activity over time, particularly as shared information and behavioral strategies may remain readily accessible following the end of such an intervention via continual access to and further engagement with previously posted content following program termination. Relative to the evaluation of social media as a form of treatment delivery, researchers should further investigate the differential influence, if any, of passive and active engagement in a virtual environment on physical activity behaviors. Additionally, new and innovative ways to increase engagement among participants should be identified and evaluated. For example, encouraging more participant/user-generated content, such as sharing photos of places to be active or creating “how to” video tutorials, may increase perceptions of ownership and accountability among participants in the program. Indeed, increased engagement by participants should lead to more effective interventions delivered via social media, and, as a result, may lead to greater program satisfaction as well as improved health-related behaviors and outcomes [51]. Last, while the *SMART* Trial chose to use Facebook to deliver this intervention, future studies may want to explore the potential of other commonly used social media services (eg, Twitter) to examine and compare their potential to promote health-related behaviors [52].

### Conclusions

The present study provides initial support for the feasibility of delivering behavioral interventions via social media [53]. The social and interactive nature of social media, along with its low cost and accessibility, make it an appealing avenue in which to target and influence health behaviors, such as physical activity. Furthermore, delivering social media-based programs can overcome many of the constraints that are commonly found with more traditional Internet- (eg, limited interactivity), print- (eg, text-heavy), and/or center-based (eg, travel) interventions. However, the effectiveness of promoting positive behavior change via social media remains to be determined (see Multimedia Appendix 1).

## Multimedia Appendix 1

PowerPoint slides for conference presentation.

## Multimedia Appendix 2

CONSORT-EHEALTH checklist V1.6.2 [54].

## Abbreviations

ANOVA: analysis of variance
BMI: body mass index
GLTEQ: Godin Leisure-Time Exercise Questionnaire
MVPA: moderate-to-vigorous physical activity
PSA: public service announcement
SMART: Social Media and Activity Research in Teens
