# Supplementary material for: Promoting Physical Activity in Low-Active Adolescents via Facebook: A Pilot Randomized Controlled Trial to Test Feasibility
Source: JMIR Res Protoc. 2014 Oct 30;3(4):e56. doi: 10.2196/resprot.3013 (PMC4259906; doi:10.2196/resprot.3013)

Using Facebook to Influence Adolescent Physical Activity:

A Pilot Randomized Controlled Trial

Thomas R. Wójcicki,
Diana Grigsby-Toussaint,
Charles H. Hillman,
Marian Huhman, &
Edward McAuley

ILLINOIS

illinois.edu

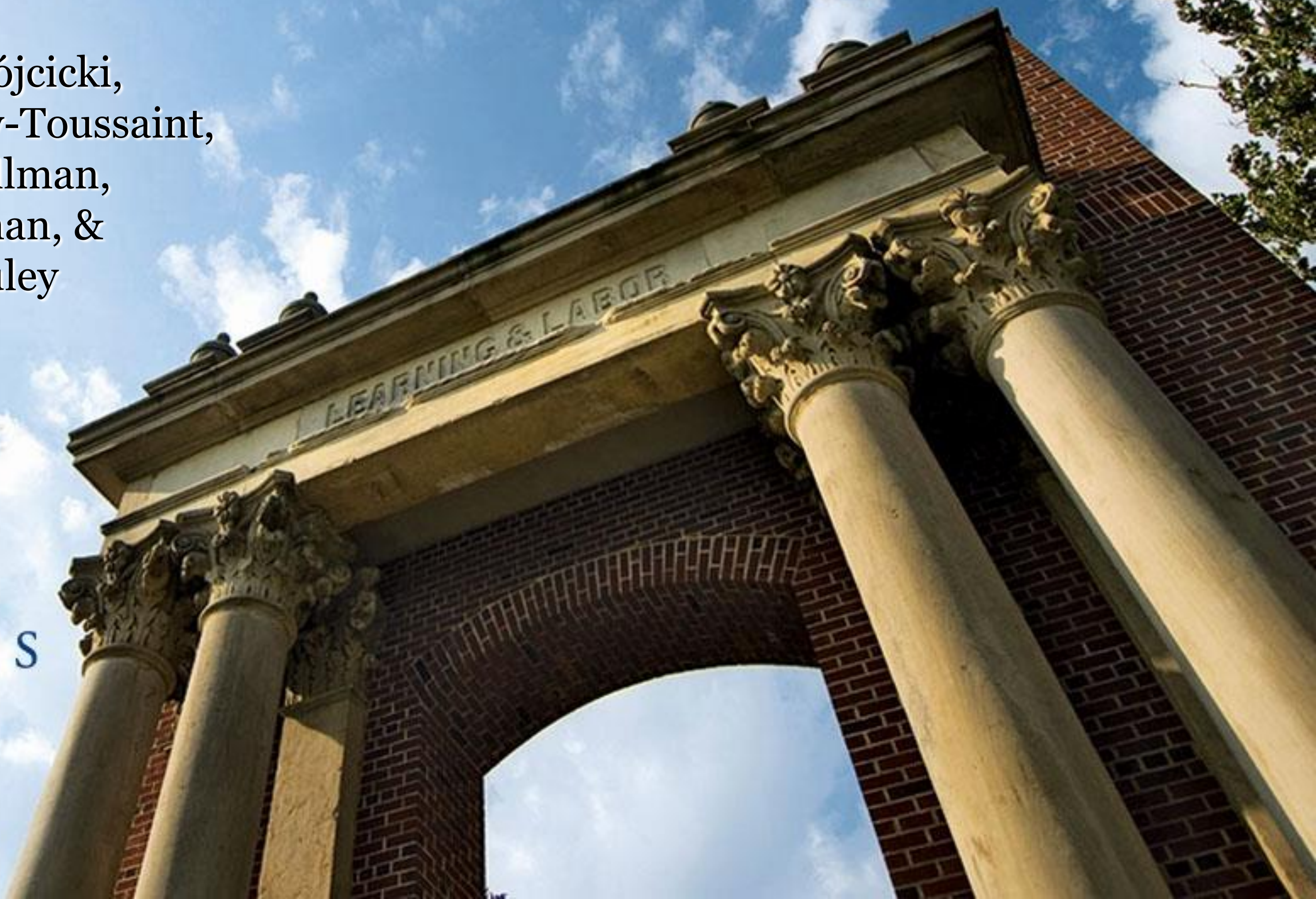

Physical Activity in Youth

- < 1/5 adolescents report being regularly physically active (CDC, 2011)
  - Active Transport
  - Physical Education
  - Sedentary Behaviors
  - Obesity Epidemic
  - Function of Age

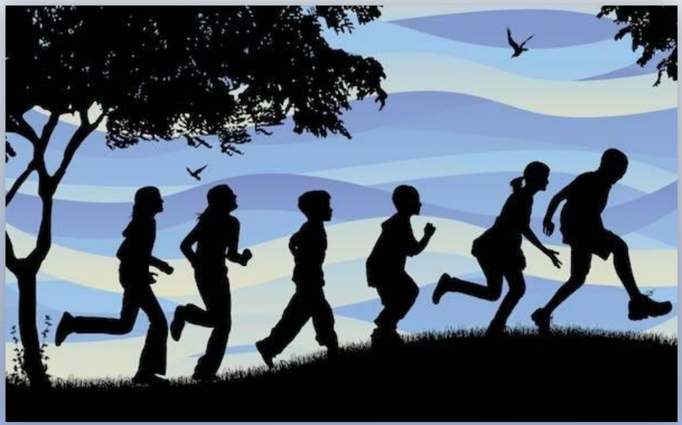

- Many health-related consequences
- Identify effective strategies to promote physical activity

Internet-Based Interventions

- Can lead to improvements in behavior change outcomes (Lau et al., 2011)
- Web 2.0
  - Socially interactive, virtual environment that fosters the creation and exchange of user-generated content
  - Has gained universal acceptance among Internet users
  - Evaluation of social media as a means to influence health behaviors is warranted (AHA, 2013; CDC, 2013; SBM, 2013)
  - Social Networking Sites are the most popular Web category
    - 57% look to their SNS for advice (e.g., health, diet, fitness) (Nielsen, 2009)

The SMART Trial

smart
social media and activity research in teens

- 8-week, randomized design PA program
  - Delivered entirely over social media
  - Goal to influence PA behaviors in sedentary and low-active adolescents

- Objectives

1. Test the feasibility of a social media-delivered PA intervention for increasing lifestyle PA
2. Compare the effects of a progressive behavioral training condition to a simple information-based condition

Hypotheses

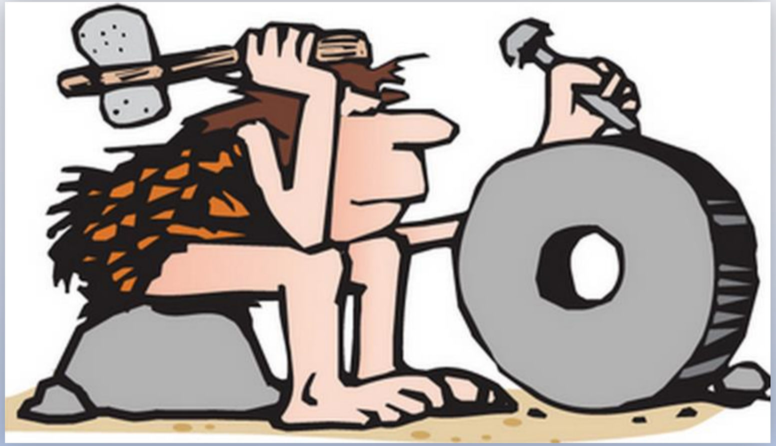

1. Trial would provide initial support for the feasibility of using social media to deliver a PA intervention

2. Behavioral training condition (i.e., intervention group) would experience greater improvements in PA

Recruitment & Eligibility

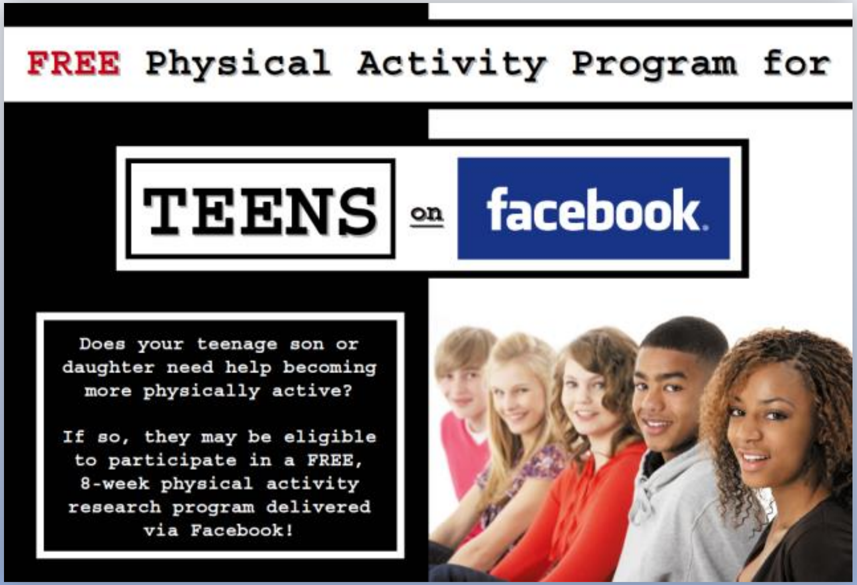

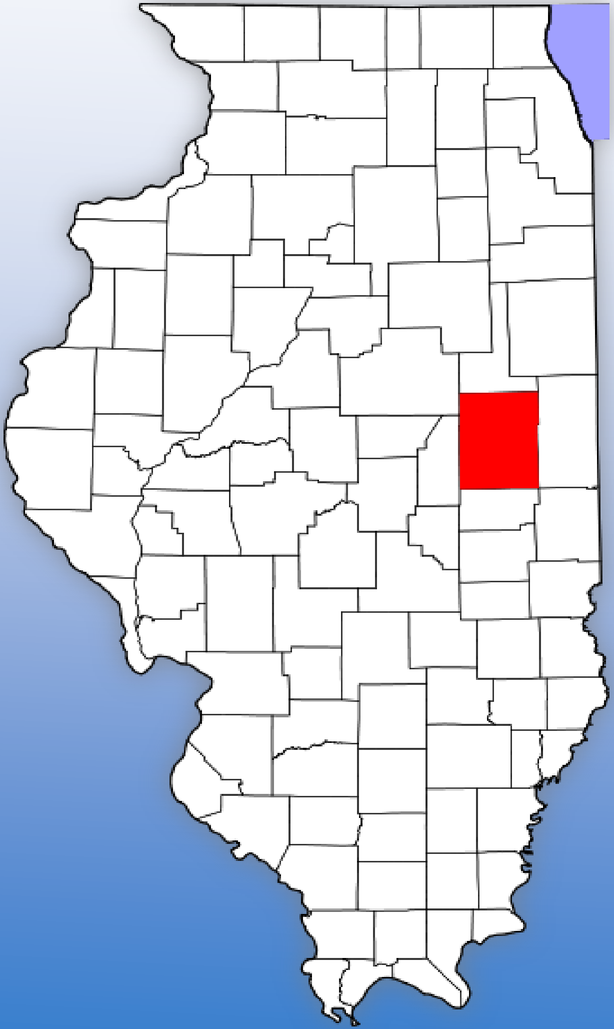

13-15 Years Old

Resident of Champaign County

< 60 Minutes of PA per Day

Internet Access, Facebook Account

N = 21 Parent-Child Dyads

Behavioral Assessments

- Conducted at baseline and program end (i.e., Week 8)
  - Accelerometry
    (Health One Technology, Fort Walton Beach, FL)
  - Godin Leisure-Time Exercise Questionnaire
    (Godin & Shephard, 1985)

Treatment Conditions

- ***Behavioral*** ***(n=10)***
  - 8 unique PA-related modules (via YouTube)
  - Privately delivered on a weekly basis via FB Messages
- ***Informational*** ***(n=11)***
  - 8 generic messages sent

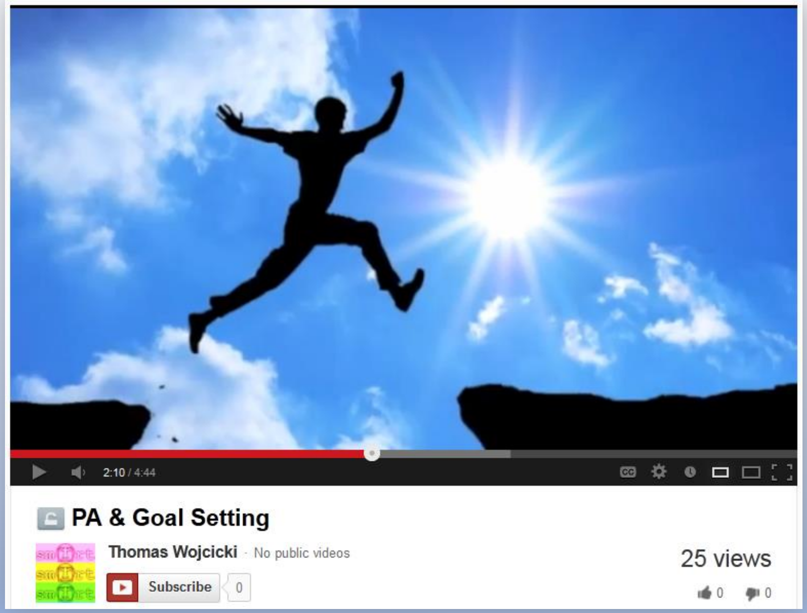

Mod1 - Getting Started with the SMART Program
Mod2 - Physical Activity Definitions and Benefits
Mod3 - Physical Activity Guidelines
Mod4 - Goal-Setting for Physical Activity
Mod5 - Individual Expectations and Physical Activity
Mod6 - Social Support for Physical Activity
Mod7 - Overcoming Barriers to Physical Activity
Mod8 - Maintaining a Physically Active Lifestyle

The SMART Facebook Group

- ***SMART FB Group***
  - Study-specific, restricted access
  - Interactive community that revolved around the topic of physical activity for adolescents

- **Two Group Posts/Day**
  1. PA-Related Websites
  2. Infographics
  3. Video PSAs
  4. Tech & Mobile Apps
  5. Local Parks & Facilities
  6. Motivational Quotes
  7. Miscellaneous

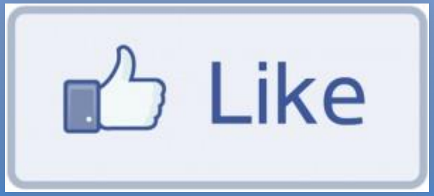

SMART Facebook Group Post

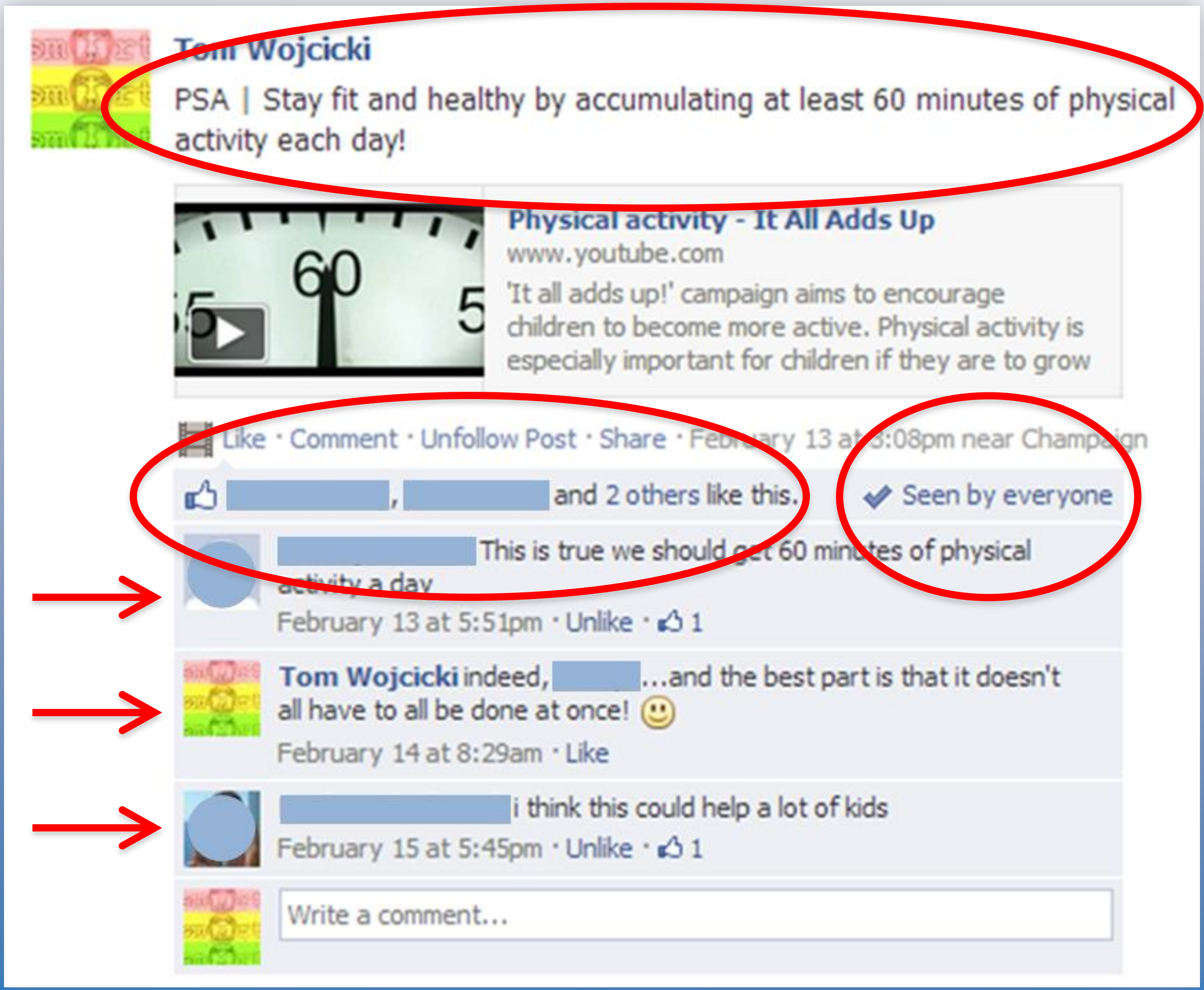

Data Analyses

- 2 (treatment condition) by 2 (time) repeated measures design

- Effect sizes (Cohen's *d*) calculated within groups to determine differential treatment effects outcomes

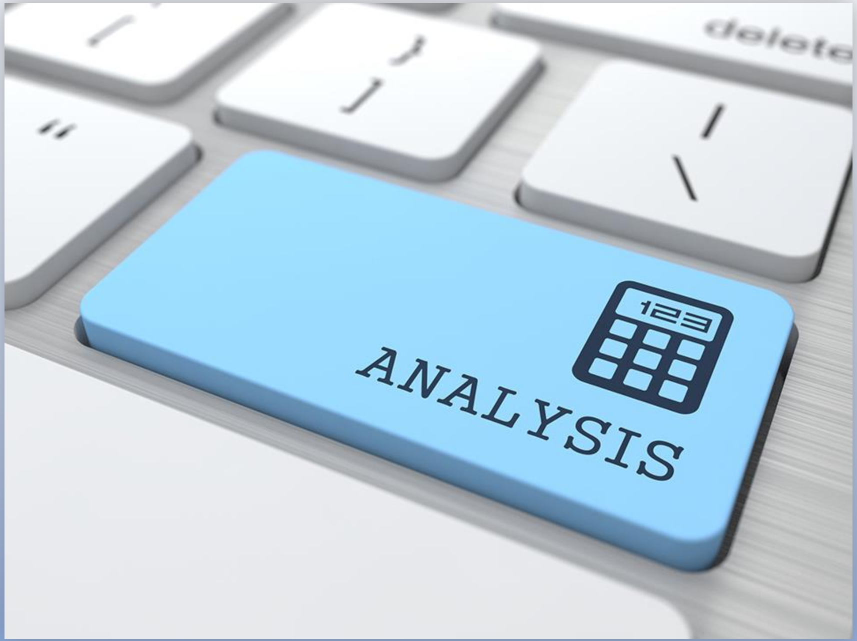

Sample Demographics

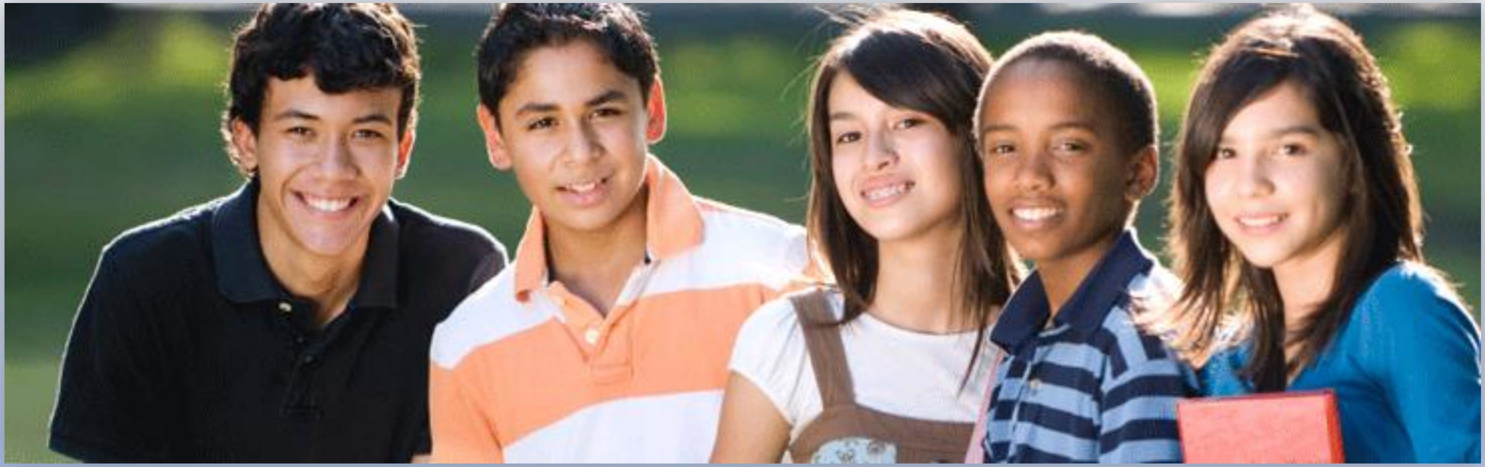

13.48 Years Old

42.90% 8th Grade

57.20% OW/Obese

61.90% White

52.40% Female

71.40% > $100K AHI

Intervention Effects

- 2x2 repeated measures design revealed improvements over time on subjectively reported:
  - Weekly leisure-time PA ($F = 8.426$, $p = .009$, $\eta^2 = .319$)
  - MVPA approached significance ($F = 4.186$, $p = .056$, $\eta^2 = .189$)
  - Significant time and/or interaction effects not present in remaining behavioral outcomes
- Effect sizes calculated to identify patterns of change within each treatment condition

Effects on Objective PA

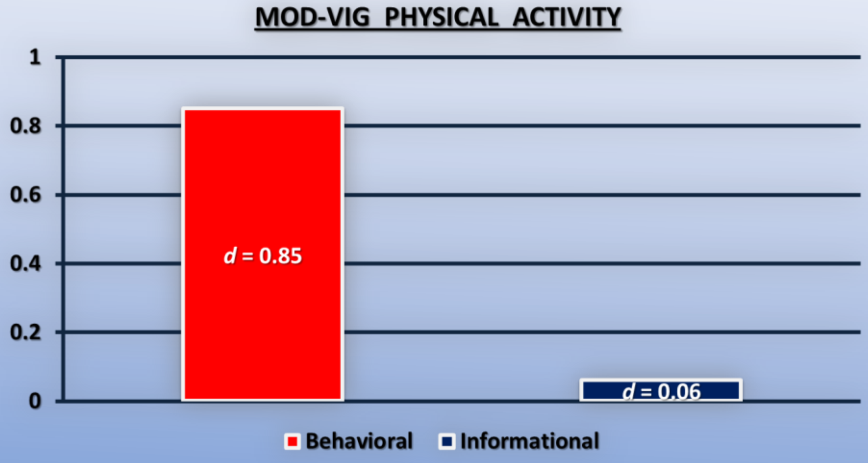

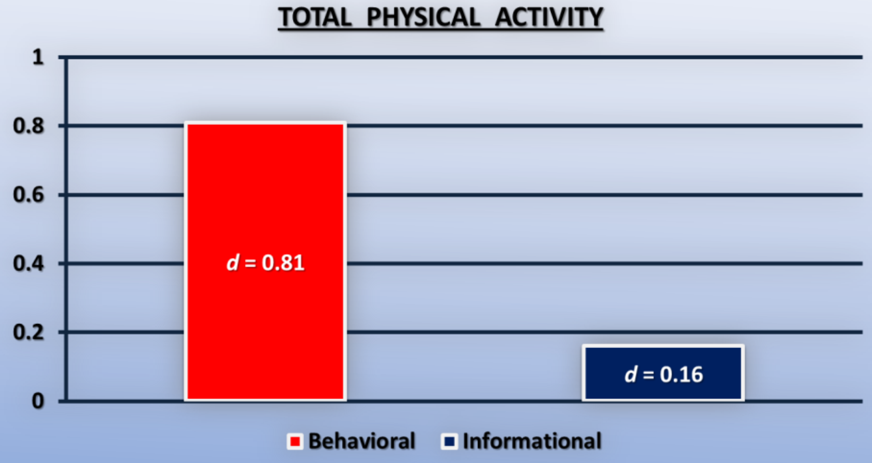

Effect Sizes

Small = .20 Medium = .50 Large = .80

Effects on Subjective PA

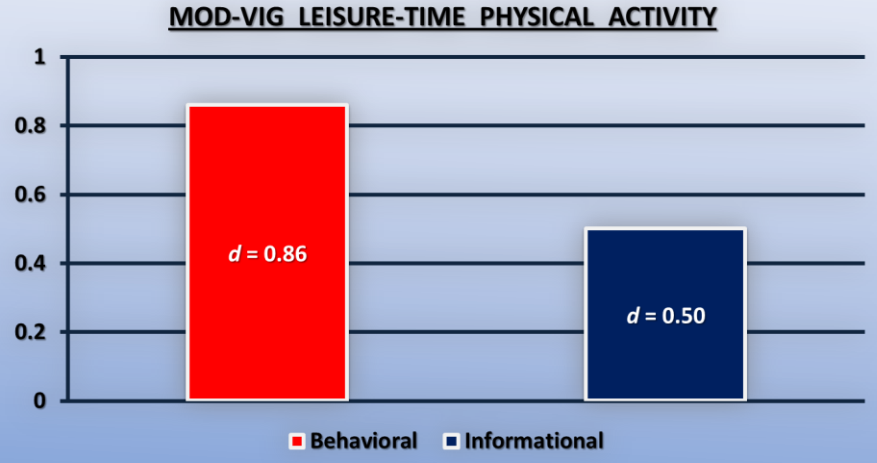

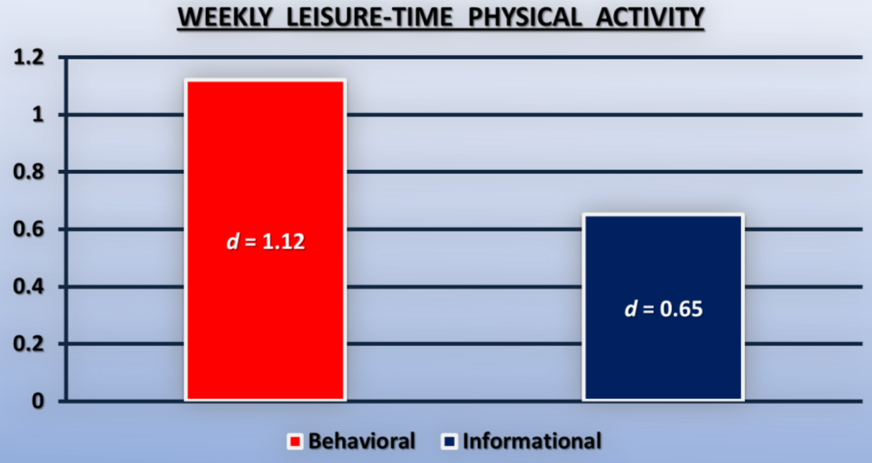

Effect Sizes

Small = .20 Medium = .50 Large = .80

Participation in FB Group

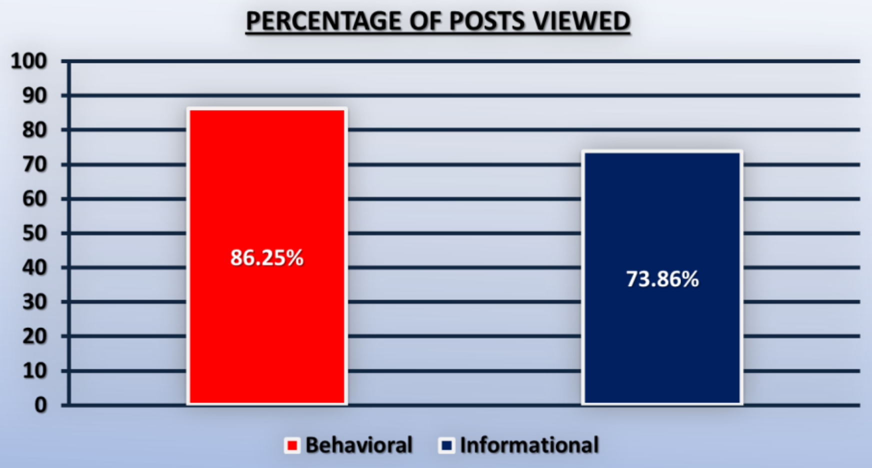

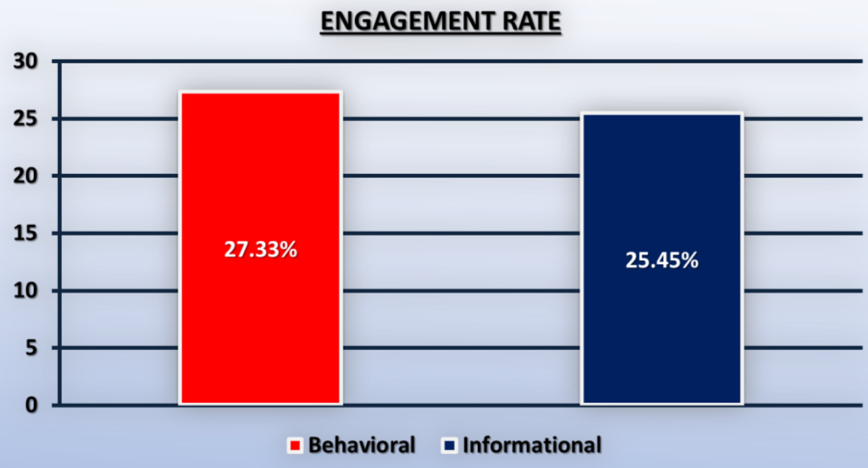

Preferred Wall Post Categories

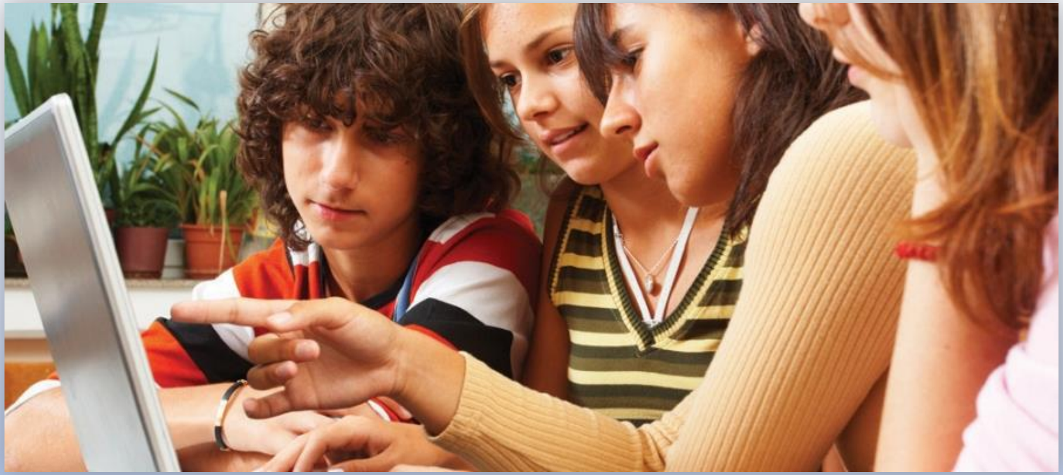

1) Technology/Apps
2) Websites
3) Miscellaneous/Topical
4) Quotations
5) Local Resources & Facilities
6) Public Service Announcements
7) Infographics

Discussion

- Changes in the behavioral outcomes were not statistically significant between groups
- Behavioral group effect sizes: moderate – large
- Informational group effect sizes: small – moderate
- Intervention effects were larger than those typically found in traditionally-delivered PA programs and web-based interventions
- Difference in behavioral effects may be specific to program delivery
  - Inherently social and interactive in nature
  - Readily available, instant access to content

Discussion

- Delivering a behavioral intervention via social media is both feasible and well-accepted

- Advances in Web 2.0 design may:
  - Influence health education and behaviors
  - Overcome many research-related barriers
  - Implications for public health

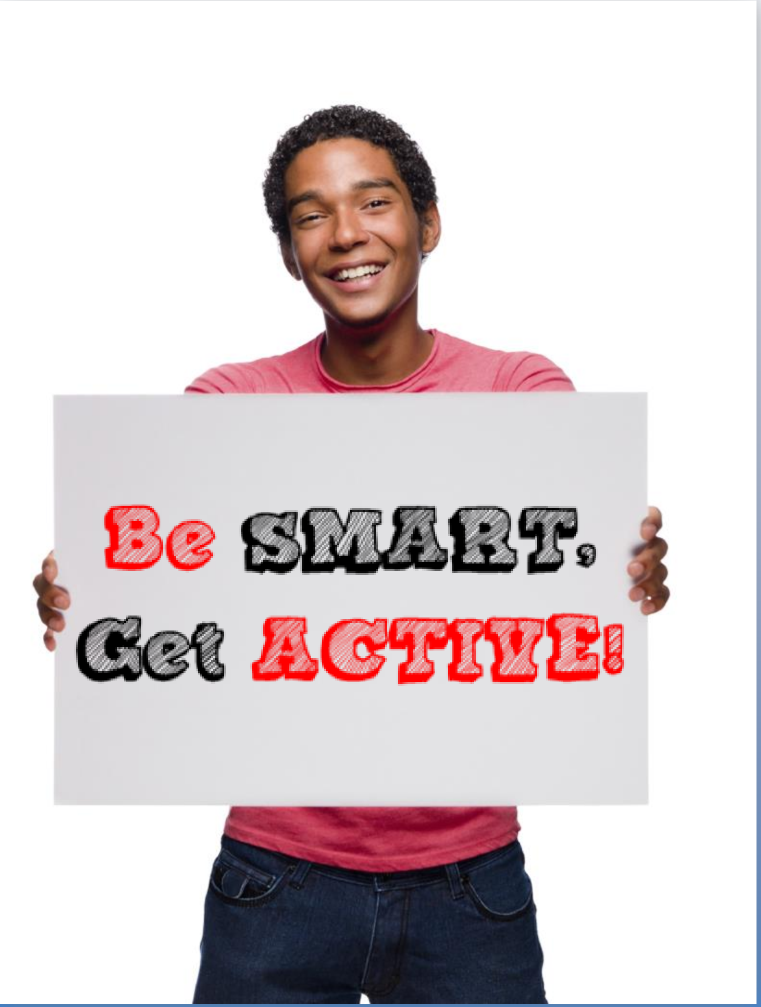

Strengths & Limitations

- Strengths
  - Among the first to examine the feasibility of delivering a PA RCT over social media
  - Subjective *and* objective assessment of behavioral outcomes
  - Fairly even split between males and females
  - ~ 40% minority participants

- Limitations
  - Small study sample, lack of statistically significant findings
  - Predominantly higher SES households
  - Inability to track the viewership of the weekly behavioral modules

Future Directions

- Larger trials to establish efficacy
- Evaluate in other populations
- Examine behavioral maintenance
- Identify strategies to increase rate of engagement with shared content
- Examine of the utility of various interactive features
- Explore the potential of other social media services

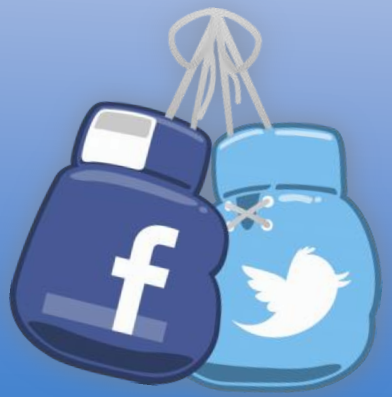

Thank You!

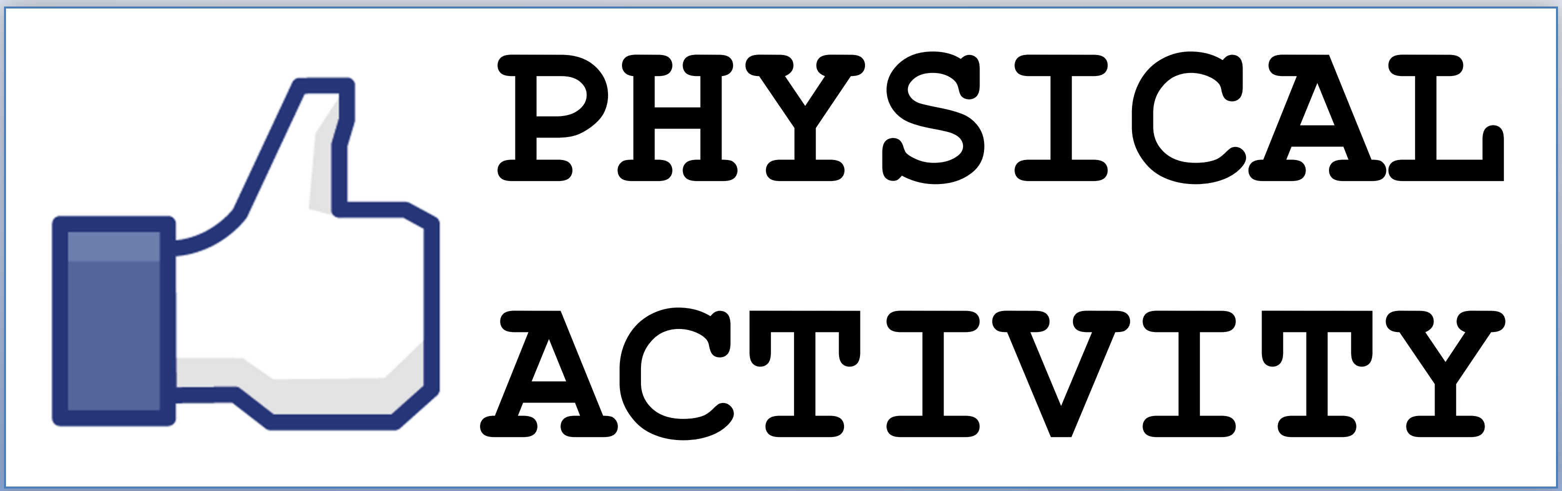

Supplement: Supplementary file 1 [file resprot_v3i4e56_app1.pdf]
