# Supplementary material for: Promoting Physical Activity in Low-Active Adolescents via Facebook: A Pilot Randomized Controlled Trial to Test Feasibility
Source: JMIR Res Protoc. 2014 Oct 30;3(4):e56. doi: 10.2196/resprot.3013 (PMC4259906; doi:10.2196/resprot.3013)
Supplement: Supplementary file 2 [file resprot_v3i4e56_app2.pdf]

CONSORT-EHEALTH Checklist V1.6.2 Report

(based on CONSORT-EHEALTH V1.6), available at [http://tinyurl.com/consort-ehealth-v1-6].

3013

Date completed
10/8/2013 12:26:32
by
Thomas Wójcicki

Facebook and Physical Activity Behavior Change in Low-Active Adolescents: A Randomized Controlled Trial

TITLE

1a-i) Identify the mode of delivery in the title
Yes

This pilot RCT was delivered entirely over Facebook; as such, the title of this manuscript highlights Facebook as the mode of delivery. Using the term "web-based" does not accurately reflect the use of Web 2.0 technologies. Furthermore, "social media-based" would not be appropriate, as the aim of this trial is to evaluate the efficacy of a specific social networking site (i.e., Facebook).

1a-ii) Non-web-based components or important co-interventions in title

1a-iii) Primary condition or target group in the title
Yes

"Low Active Adolescents"

ABSTRACT

1b-i) Key features/functionalities/components of the intervention and comparator in the METHODS section of the ABSTRACT
Yes

"Participants were randomized to either a social cognitive-based condition (i.e., Behavioral group) or an attentional control (i.e., Informational group). Both conditions received access to a restricted-access, study-specific Facebook Group where two daily Wall Posts containing youth-based physical activity information and resources were made by the Group's administrator."

1b-ii) Level of human involvement in the METHODS section of the ABSTRACT

1b-iii) Open vs. closed, web-based (self-assessment) vs. face-to-face assessments in the METHODS section of the ABSTRACT

1b-iv) RESULTS section in abstract must contain use data

1b-v) CONCLUSIONS/DISCUSSION in abstract for negative trials

INTRODUCTION

2a-i) Problem and the type of system/solution
Yes

"Traditionally, physical activity interventions for youth have often used in-person, center-based modes of delivery [16,17]. Although these face-to-face interventions have been identified as the "gold standard" of behavioral therapy, they are limited in terms of reach and accessibility. Fortunately, the relatively recent introduction of the Internet into clinical practice has created several opportunities for innovative behavioral interventions [18,19]. Researchers have become increasingly more interested in using the Internet as a mode of delivery for physical activity programs [17,20], and a number of reviews attest to the feasibility and efficacy of Internet-delivered physical activity interventions for changing health behavior outcomes [21-23]."

2a-ii) Scientific background, rationale: What is known about the (type of) system
Yes

This was an efficacy trial (the first of its kind); therefore, comparisons with other interventions could not be made. However, literature regarding the use and potential have been cited.

"More recently, advances in web-based programing have led to interactive communication technologies, commonly referred to as Web 2.0. These sites provide users with the ability to make virtual connections and interact with one another in a social media dialogue based on user-generated content [24-26]. Due, in part, to the ease of use, accessibility, minimal cost, limited maintenance (on the user's end), and various interactive features, these relatively new technologies have quickly gained universal acceptance. As a result, researchers have begun to examine the potential of social media as a means for increasing education and social support for individual behavior change. Emerging evidence suggests that physical activity interventions using social media can be effective at influencing health and promoting behavior change [23,27,28]. Innovative Web 2.0 interventions have fewer limitations (e.g., reach- and accessibility-related issues, cost of development and maintenance, time required) than the more traditional Internet-delivered behavioral interventions (e.g., static websites, e-mail). Provided with inherent interactive communication features, social media sites provide users with the ability for continuous self-monitoring, real-time feedback, and information exchange, all of which are conducive to behavior change [19]. Thus, further evaluation of social media as a means to influence health behaviors is warranted [29-31]."

METHODS

3a) CONSORT: Description of trial design (such as parallel, factorial) including allocation ratio
Yes

"The Social Media and Activity Research in Teens Trial (i.e., SMART Trial) is a social media-based intervention specifically designed to influence the physical activity behaviors of adolescents. Based on social cognitive principles and with physical activity as the primary outcome of interest, this innovative eight-week behavioral program was delivered entirely through a social networking site (i.e., Facebook). The primary objectives of this trial were to: 1) test the efficacy of a social media-delivered physical activity intervention for increasing lifestyle physical activity in adolescents; and 2) determine the effectiveness of a physical activity intervention based on social cognitive principles in comparison to a simple information-based physical activity condition. It was hypothesized that exposure to and participation in this social media-delivered intervention would be efficacious in promoting and producing physical activity changes in adolescents over the course of the intervention. Additionally, involvement in a social cognitive-based intervention group should lead to greater improvements in physical activity participation and related psychosocial constructs in comparison to an attentional control group."

3b) CONSORT: Important changes to methods after trial commencement (such as eligibility criteria), with reasons
No changes were made to the methods after trial commencement.
3b-i) Bug fixes, Downtimes, Content Changes

4a) CONSORT: Eligibility criteria for participants
Yes

"To be considered for participation a parent or legal guardian had to accompany all eligible adolescent participants, aged 13-15 years. At screening, parents of potential participants were asked to estimate their child's average weekly physical activity levels, allowing for the identification and, as a result, exclusion of those meeting or exceeding the current physical activity guidelines. Additionally, participants were required to be English-speaking and have access to the Internet at their place of residence via a personal or family-dedicated tablet, laptop, or desktop computer. Individuals who only had mobile access to the Internet were excluded. Finally, participants also had to have an active Facebook account or be willing to create one prior to enrollment."
4a-i) Computer / Internet literacy

4a-ii) Open vs. closed, web-based vs. face-to-face assessments:
Yes

"We used targeted mailings via the United States Postal Service (i.e., USPS), the Internet (e.g., e-mail listservs, social media). Given that this trial relied heavily on parental involvement, recruitment efforts were targeted at parents or legal guardians of children between the ages of 13 and 15 years. Recruitment ads included brief and basic information about the study, along with contact information (i.e., study-specific e-mail and website) for interested persons."
4a-iii) Information giving during recruitment

4b) CONSORT: Settings and locations where the data were collected
Yes

"Physical and behavioral assessments were conducted at baseline and at the end of the trial (i.e., Week 8). Demographics, anthropomorphic measures, and health history were conducted onsite. Physical activity was assessed subjectively, using self-report, and objectively, using an activity monitor. All self-report assessments were administered electronically via a web-based survey service (i.e., SurveyMonkey; Palo Alto, CA, 2012). When appropriate, participants received a web link to a battery of questionnaires via Facebook's direct messaging service. Submitted data was unidentifiable and stored in a secure, password protected online database."
4b-i) Report if outcomes were (self-)assessed through online questionnaires
Yes

"During their baseline visit to the Exercise Psychology Laboratory, participants and at least one of their legal guardians were added to the private SMART Facebook Group via an invitation from the Group's administrator. Participants and their guardians had to first accept a Friend Request from the administrator in order to be electronically invited to join the SMART Group. To ensure confidentiality and improve experimental rigor, this group was kept private to the randomized child-parent pairs. Once accepted to the SMART Group, all participants, regardless of treatment allocation, were sent privately delivered (i.e., using Facebook Messages), secure links to complete the web-based battery of questionnaires, on their own time and prior to the official start date of the trial."

"The baseline laboratory visit also included anthropometric assessments of study participants via a digital scale and stadiometer. Once this information was obtained, participants were provided with an accelerometer and were asked to mail the device, along with a corresponding accelerometer log to validate wear time, in a pre-addressed, self-stamped envelope. Finally, prior to leaving the baseline appointment, all participants, regardless of group allocation, were given pedometers to use over the course of the trial to aid in self-monitoring and behavioral regulation."
4b-ii) Report how institutional affiliations are displayed

5) CONSORT: Describe the interventions for each group with sufficient details to allow replication, including how and when they were actually administered
5-i) Mention names, credential, affiliations of the developers, sponsors, and owners

5-ii) Describe the history/development process

5-iii) Revisions and updating

5-iv) Quality assurance methods

5-v) Ensure replicability by publishing the source code, and/or providing screenshots/screen-capture video, and/or providing flowcharts of the algorithms used

5-vi) Digital preservation

5-vii) Access

Yes

"Access to the SMART Facebook Group was restricted to study participants and at least one of their legal guardians. The purpose of this Group was to create a social, interactive community that revolved around the topic of physical activity for youth. Reliable physical activity information and resources from around the Web were provided daily by the group's administrator. Considering the group-based characteristics and preferences, an assortment of Wall Posts were made, all of which were categorized within one of the following seven categories: 1) physical activity-related websites; 2) infographics; 3) public service announcements; 4) technology and applications; 5) local parks and facilities; 6) motivational quotations; and 7) miscellaneous topical posts. The SMART Group received two Wall Posts per day (i.e., once in the morning and again in the evening), resulting in 14 total Wall Posts per week with equal distribution of the pre-defined content categories. In addition to these daily posts, photo albums containing physical activity campaign advertisements/posters (e.g., The President's Challenge) were uploaded and shared once per week, resulting eight additional Posts. In all, 120 Posts were made over the course of the trial. Recommended strategies for effective wall posts (i.e., posts that encourage group-member engagement via Likes and Comments) were utilized throughout the eight-week program [52]."

Also, see Table 1.

5-viii) Mode of delivery, features/functionalities/components of the intervention and comparator, and the theoretical framework

Yes

Behavioral Condition

"Participants in the Behavioral condition received full access to content posted on the SMART Group Wall and were encouraged to regularly view and interact with (i.e., Like, Comment, and Share) the Posts. In addition to Group access, participants in the treatment condition regularly received social cognitive-based content (i.e., Behavioral Modules) via Facebook Messages in the form of 5-10 minute YouTube videos created by the Group administrator and co-principal investigator. The SMART Group administrator was responsible for distributing this information to each participant and followed a pre-determined schedule (i.e., the first day of each intervention week). Over the course of the intervention, the Group administrator delivered eight unique physical activity modules based on social cognitive principles (i.e., self-efficacy, outcome expectations, goals, and sociocultural factors) to help influence behavior change among the adolescent participants. Beginning with the start of the intervention, these Behavioral Modules were privately delivered (i.e., content can only be viewed by the sender and recipient), via a restricted-access YouTube link, to each participant and corresponding guardian every Monday morning. These messages always included a personalized greeting, written information regarding the content of the video-based module, and a prompt encouraging participants to ask the Group administrator questions relative to the modules' content, should they have any (to access these modules, see Table 1 for tiny URLs)."

Informational Condition

"Participants in the Informational condition also received full access to the SMART Group and were similarly encouraged to regularly view and interact with the content posted on the Group Wall. Informational participants were also be contacted once a week by the Group administrator via a private Facebook Message. The frequency of theses fairly generic yet personalized messages (e.g., "Hey Johnny! We are entering the 4th week of the program. Thank you for your commitment and remember to visit the SMART group daily and to interact with the Wall Posts") occurred once per week – in unison with the delivery of the Behavioral Modules."

5-ix) Describe use parameters

5-x) Clarify the level of human involvement

5-xi) Report any prompts/reminders used

Yes

Daily posts in Facebook Group and weekly private Facebook messages to each parent-child dyad.

5-xii) Describe any co-interventions (incl. training/support)

Co-interventions were not present in this study.

6a) CONSORT: Completely defined pre-specified primary and secondary outcome measures, including how and when they were assessed

Yes

Objective Physical Activity

"Accelerometry was used to objectively assess participants' physical activity levels. Specifically, the rechargeable, lithium-powered Actigraph accelerometer (models GT1M and GT3X; Health One Technology, Fort Walton Beach, FL) was used for this purpose, as it is the most commonly used accelerometer in the field of physical activity-related research [44] and has been shown to provide reliable and valid estimates of energy expenditure and activity levels in youth [45-47]. Participants were instructed to wear the accelerometer on their non-dominant hip during all waking hours (with the exception of water based activities) for seven consecutive days, as recommended by Trost and colleagues [48]. Written instructions detailing proper use and an accelerometer log to verify wear time were also provided to the study participants. Following pre-programmed initialization, the monitor began to collect daily activity counts on a 30-second basis (i.e., epochs). Upon completion of requested wear time and receipt of the device via the USPS, these data were downloaded, summed, and divided by seven (or number of days worn) to provide an objective measure of the participants' average daily physical activity levels. Recommended cut points for predicting physical activity in youth were utilized to properly identify the amount of engagement in sedentary, light, moderate, and vigorous activities [44]."

Subjective Physical Activity

"Self-reported involvement in physical activities was collected and assessed using the Godin Leisure Time Exercise Questionnaire (GLTEQ) [49]. The GLTEQ is a well-validated brief assessment of usual leisure-time exercise habits [50]. The first, three-part question asks participants to report how many times they participate in strenuous (i.e., heart beats rapidly), moderate (i.e., not exhausting), and mild (i.e., minimal effort) activities for more than 15 minutes over the course of a typical week (i.e., 7-day period). The reported frequencies of strenuous, moderate, and mild activities are multiplied by 9, 5, and 3 metabolic equivalents, respectively, and then summed to provide a reliable estimate of total weekly leisure time activity."

6a-i) Online questionnaires: describe if they were validated for online use and apply CHERRIES items to describe how the questionnaires were designed/deployed

6a-ii) Describe whether and how "use" (including intensity of use/dosage) was defined/measured/monitored

6a-iii) Describe whether, how, and when qualitative feedback from participants was obtained

6b) CONSORT: Any changes to trial outcomes after the trial commenced, with reasons
There were no changes to trial outcome after the trial commenced.
7a) CONSORT: How sample size was determined
7a-i) Describe whether and how expected attrition was taken into account when calculating the sample size

7b) CONSORT: When applicable, explanation of any interim analyses and stopping guidelines
Interim analyses and stopping guidelines were not utilized/conducted during this trial.
8a) CONSORT: Method used to generate the random allocation sequence
Yes

"Of these 33 contacts, 21 participants met the inclusion criteria and were randomized by sex and age into one of the two treatment conditions via SPSS version 22 (SPSS IBM, New York, USA)."
8b) CONSORT: Type of randomisation; details of any restriction (such as blocking and block size)
Randomized by sex and age.
9) CONSORT: Mechanism used to implement the random allocation sequence (such as sequentially numbered containers), describing any steps taken to conceal the sequence until interventions were assigned
SPSS version 22 (SPSS IBM, New York, USA)
10) CONSORT: Who generated the random allocation sequence, who enrolled participants, and who assigned participants to interventions
Thomas Wójcicki and Edward McAuley
11a) CONSORT: Blinding - If done, who was blinded after assignment to interventions (for example, participants, care providers, those assessing outcomes) and how
11a-i) Specify who was blinded, and who wasn't
Neither Thomas Wójcicki (responsible for delivering intervention) nor the participants (clearly received more or less information - e.g., access to weekly behavioral modules via YouTube) were blinded. Co-authors were blinded.
11a-ii) Discuss e.g., whether participants knew which intervention was the "intervention of interest" and which one was the "comparator"

11b) CONSORT: If relevant, description of the similarity of interventions
No - not relevant
12a) CONSORT: Statistical methods used to compare groups for primary and secondary outcomes
Yes

"Data were checked for missing items, normality, outliers, and errors. A series of independent-samples t tests were conducted to identify significant differences in demographic data and descriptive statistics of study variables. Using an intent-to-treat approach, the efficacy of the intervention in producing behavioral and psychosocial changes was examined using a 2 (condition: Behavioral vs. Informational group) by 2 (time) repeated measures design from data collected at baseline and at the end of the intervention. Interactions and main effects were examined, as well as mean differences in effect sizes. Given the pilot nature of this trial and the importance of adequately powering subsequent larger trials, effect sizes (Cohen's d) were also calculated within groups to further determine differential treatment effects on study outcome variables."
12a-i) Imputation techniques to deal with attrition / missing values
Intent-to-treat analysis
12b) CONSORT: Methods for additional analyses, such as subgroup analyses and adjusted analyses
Yes

"Data were checked for missing items, normality, outliers, and errors. A series of independent-samples t tests were conducted to identify significant differences in demographic data and descriptive statistics of study variables. Using an intent-to-treat approach, the efficacy of the intervention in producing behavioral and psychosocial changes was examined using a 2 (condition: Behavioral vs. Informational group) by 2 (time) repeated measures design from data collected at baseline and at the end of the intervention. Interactions and main effects were examined, as well as mean differences in effect sizes. Given the pilot nature of this trial and the importance of adequately powering subsequent larger trials, effect sizes (Cohen's d) were also calculated within groups to further determine differential treatment effects on study outcome variables."

RESULTS

13a) CONSORT: For each group, the numbers of participants who were randomly assigned, received intended treatment, and were analysed for the primary outcome
Yes

"Two separate rounds of recruitment occurred, which resulted in two waves of participants for the trial. In total, 33 contacts were made and screened for eligibility. Of these 33 contacts, 21 participants met the inclusion criteria and were randomized by sex and age into one of the two treatment conditions via SPSS version 22 (SPSS IBM, New York, USA). Considering the recruitment methods that were employed, the targeted mailing of recruitment post cards had both the largest reach (i.e., approximately 3,200 households throughout Champaign County) and highest rate of return with 61.90% of study participants responding to these advertisements. This was followed by mass e-mails to various listservs (28.60%); announcements via the Champaign Public Health District, as well as the one-time delivery of a weekly digital university newsletter each resulted in attaining 4.80% of the sample. Of the 21 randomized participants, 20 completed assessments at both baseline and follow-up, resulting in 4.76% rate of attrition. Additional details regarding participant flow within the SMART Trial can be found in Figure 1."

13b) CONSORT: For each group, losses and exclusions after randomisation, together with reasons
Yes

See CONSORT diagram near end of manuscript
13b-i) Attrition diagram

14a) CONSORT: Dates defining the periods of recruitment and follow-up
Yes

"Physical and behavioral assessments were conducted at baseline and at the end of the trial (i.e., Week 8)."
14a-i) Indicate if critical "secular events" fell into the study period

14b) CONSORT: Why the trial ended or was stopped (early)
Not applicable
15) CONSORT: A table showing baseline demographic and clinical characteristics for each group
Yes

See Tables section of manuscript.
15-i) Report demographics associated with digital divide issues
No - as a requirement for entry to the study, all participants needed to have access to a home-based computer with Internet connection.
16a) CONSORT: For each group, number of participants (denominator) included in each analysis and whether the analysis was by original assigned groups
16-i) Report multiple "denominators" and provide definitions
Sample sizes for pre-post testing are provided throughout the results section and can also be found within the Tables section. All analyses were by original assigned groups.
16-ii) Primary analysis should be intent-to-treat

17a) CONSORT: For each primary and secondary outcome, results for each group, and the estimated effect size and its precision (such as 95% confidence interval)
Yes

"Given the small sample size of this trial, effect sizes were calculated to identify the patterns of change within each treatment group and to better quantify the effectiveness of the intervention. Mean values for all physical activity and sedentary outcomes as well as effect sizes for the total sample and each condition are reported in Tables 5 – 7. As can be seen from the accelerometer data, the Behavioral group clearly increased their activity levels across the trial relative to the Informational group. In terms of minutes of physical activity per day, involvement in the Behavioral condition produced a medium effect (d = 0.52), as compared to a small effect (d = 0.24) in the Informational condition. Additionally, improvements were found for moderate-to-vigorous (d = 0.85) and total physical activity (d = 0.81) in the Behavioral group, whereas the effect sizes for the Informational group reflected little change (d = 0.06 and 0.16, respectively). Effect sizes for objectively assessed sedentary counts were similar between the intervention (d = -0.02) and comparison (d = -0.03) groups. Effect sizes indicated substantially greater improvements in subjectively assessed physical activity for the Behavioral group compared to the Informational group. Large and positive effects were found for both moderate-to-vigorous (d = 0.86) and weekly leisure-time (d = 1.12) physical activities in the Behavioral group, although participants in the Informational group also reported moderate increases (d = 0.50 and 0.65, respectively). The effect sizes for subjectively assessed sedentary behaviors, on the other hand, were small to non-existent for both conditions (d = 0.15, Behavioral group; d = -0.06, Informational group)."
17a-i) Presentation of process outcomes such as metrics of use and intensity of use

17b) CONSORT: For binary outcomes, presentation of both absolute and relative effect sizes is recommended
NA
18) CONSORT: Results of any other analyses performed, including subgroup analyses and adjusted analyses, distinguishing pre-specified from exploratory
Yes

"Participants were asked to anonymously complete a program evaluation and feedback form that was designed specifically for this trial. Twenty of the 21 participants completed these forms at their follow-up appointment. Results revealed that 70.0% of the study sample was "satisfied" to "very satisfied" with their overall experience with the SMART Trial. Additionally, 55.0% of the sample found the content posted on the SMART Group Wall to be "interesting" to "very interesting," and 45.0% found the posted content to be "useful" to "very useful." On average, 55.0% of the participants visited the SMART Group "1 – 2 times per week," while 45.0% interacted with (i.e., Like, Comment, and/or Share) the posted content "1 – 2 times per week," as well. Furthermore, 50.0% of those surveyed indicated that they learned "a good amount" to "a great deal/a lot" about physical activity from their involvement in this program, and 70.0% felt that their participation in the program influenced their physical activity "some" to "a good amount." And finally, when asked to rate their experiences with study staff (over Facebook and in person), 95.0% of the participants reported having "good" to "excellent" interactions. Mean values for these evaluative responses are reported in Table 8. Insight into participants' preferred categories of Wall Posts was also assessed, with the Technology/App category being the most preferred type of shared content, while Infographics were rated as the least preferred category (Table 9)."
18-i) Subgroup analysis of comparing only users

19) CONSORT: All important harms or unintended effects in each group
None reported
19-i) Include privacy breaches, technical problems

19-ii) Include qualitative feedback from participants or observations from staff/researchers

DISCUSSION
20) CONSORT: Trial limitations, addressing sources of potential bias, imprecision, multiplicity of analyses
20-i) Typical limitations in ehealth trials
Yes

"Several limitations with this trial should be considered when interpreting the results and building upon the findings. First, despite considerable efforts in trying to recruit participants for this trial, the study sample was relatively small. A larger sample size would improve the power of the study and potentially reduce the amount of variance among study variables. Based on the effect size found in the Behavioral group (d = 0.85), subsequent trials would need a sample of at least 74 participants to achieve statistical significance in objectively assessed moderate-vigorous physical activity. Another limitation is the fact that a majority of the sample came from higher socioeconomically status households. Whether or not similar results would be found in participants from lower socioeconomic households remains to be determined. A further limitation was the lack of ability to track the viewership of the weekly behavioral modules by study participants. Insight into participant viewership would provide further support for the effectiveness of the Behavioral group, as the receipt of these social cognitive-based modules was the only difference between the two treatment conditions in terms of program delivery. If participants in the Behavioral condition did, in fact, view these modules on a regular basis, a more concrete conclusion could be made regarding its overall utility and overall worth in improving physical activity levels and related psychosocial outcomes."
21) CONSORT: Generalisability (external validity, applicability) of the trial findings
21-i) Generalizability to other populations

21-ii) Discuss if there were elements in the RCT that would be different in a routine application setting

22) CONSORT: Interpretation consistent with results, balancing benefits and harms, and considering other relevant evidence
22-i) Restate study questions and summarize the answers suggested by the data, starting with primary outcomes and process outcomes (use)
Yes

"Involvement in the SMART Trial resulted in small increases in objectively assessed moderate-to-vigorous and total physical activity, as well as time spent being physically active. Similarly, subjectively assessed physical activity also resulted in improvements, but the effects were substantially larger for moderate-to-vigorous physical activity and total weekly leisure-time physical activity. At first, the differences in the degree of the effects found between the objective versus subjective measures may seem odd, as both forms of measurement are intended to capture one's level of physical activity. It is important to note that the Godin Leisure-Time Exercise Questionnaire was specifically designed to assess leisure-time physical activity behaviors [49], whereas the accelerometer assesses all physical activity accumulated throughout the day, regardless of activity type. Therefore, participation in this trial appeared to be more beneficial for influencing leisure-time physical activities (e.g., going to the park, dancing, walking the dog, etc.) than for total daily physical activities (e.g., getting ready for school, walking to and from classes, doing chores, etc.).
Although changes in physical activity were not significant between groups using inferential statistics, the effect sizes that were produced in the Behavioral group were, in most cases, moderate to large in size and in the hypothesized direction, whereas the effect sizes in the Informational group were mostly small to moderate. In the case of objectively and subjectively assessed physical activity outcomes, the experimental condition experienced large increases in both moderate-vigorous and total physical activity. Involvement in the comparison group resulted in small but positive changes for objective moderate-to-vigorous and total physical activities, and moderate improvements in subjective moderate-to-vigorous and weekly leisure-time physical activity. Finally, differences among the self-reported sedentary outcomes (i.e., average weekday and weekend) were small to nonexistent in both groups. This is not entirely surprising, however, as the purpose of this trial was not aimed at minimizing sedentary behaviors, but, rather, increasing regular participation in physical activities.
The effects found in the Behavioral group were larger than the effect sizes that are typically reported in the literature for physical activity-related outcomes as a result of intervention. For example, Kamath and colleagues conducted a meta-analysis on behavioral interventions aimed at improving physical activity levels in children and adolescents [53] noting that youth-based physical activity interventions typically result in a significant but small effect ($d = 0.12$; range: 0.04 – 0.20). Moreover, Lau and colleagues conducted a review of information and communication technology-based interventions and also found small effect sizes, ranging from 0.03 to 0.41, always favoring the intervention to control conditions [26]. Importantly, none of the interventions in either of these reviews were entirely delivered via social media. Perhaps the social and interactive nature of the SMART Trial, in conjunction with the weekly receipt and viewing of the video-based behavioral modules, may provide some insight as to why this pilot intervention appears to be more effective than those that have preceded it."
22-ii) Highlight unanswered new questions, suggest future research

Other information

23) CONSORT: Registration number and name of trial registry
Yes

"ClinicalTrials.gov Identifier: NCT01870323"
24) CONSORT: Where the full trial protocol can be accessed, if available
Yes

"ClinicalTrials.gov Identifier: NCT01870323"
25) CONSORT: Sources of funding and other support (such as supply of drugs), role of funders
None Declared
X26-i) Comment on ethics committee approval

x26-ii) Outline informed consent procedures

X26-iii) Safety and security procedures

X27-i) State the relation of the study team towards the system being evaluated
None Declared
